# Synthetic lethality of PARP and NAMPT inhibition in triple-negative breast cancer cells

**DOI:** 10.1002/emmm.201201250

**Published:** 2012-08-30

**Authors:** Ilirjana Bajrami, Asha Kigozi, Antoinette Van Weverwijk, Rachel Brough, Jessica Frankum, Christopher J Lord, Alan Ashworth

**Affiliations:** 1 The Breakthrough Breast Cancer Research Centre, The Institute of Cancer ResearchLondon, UK; 2 Cancer Research UK Gene Function Laboratory, The Institute of Cancer ResearchLondon, UK

**Keywords:** breast cancer, NAMPT, PARP inhibitor, triple negative, β-NAD^+^

## Abstract

PARP inhibitors have been proposed as a potential targeted therapy for patients with triple-negative (ER-, PR-, HER2-negative) breast cancers. However, it is as yet unclear as to whether single agent or combination therapy using PARP inhibitors would be most beneficial. To better understand the mechanisms that determine the response to PARP inhibitors, we investigated whether enzymes involved in metabolism of the PARP substrate, β-NAD^+^, might alter the response to a clinical PARP inhibitor. Using an olaparib sensitization screen in a triple-negative (TN) breast cancer model, we identified nicotinamide phosphoribosyltransferase (NAMPT) as a non-redundant modifier of olaparib response. NAMPT is a rate-limiting enzyme involved in the generation of the PARP substrate β-NAD^+^ and the suppression of β-NAD^+^ levels by NAMPT inhibition most likely explains these observations. Importantly, the combination of a NAMPT small molecule inhibitor, FK866, with olaparib inhibited TN breast tumour growth *in vivo* to a greater extent than either single agent alone suggesting that assessing NAMPT/PARP inhibitor combinations for the treatment of TN breast cancer may be warranted.

## INTRODUCTION

The addition of poly(ADP-ribose) chains onto proteins (PARsylation) is a post-translational modification that has been implicated in a wide range of biological processes as diverse as the maintenance of genomic stability (Hottiger et al, 2010) and Wnt signalling (Callow et al, 2011; Huang et al, 2009; Zhang et al, 2011). PARsylation is catalysed by a series of structurally related proteins, the poly(ADP) ribose polymerase (PARP) superfamily. PARP enzymes add ADP-ribose moieties onto proteins, using β-NAD^+^ as substrate. Of the 17 or so PARP superfamily members, PARP1 is the most studied, and is known, amongst other functions, to play a critical role in DNA repair. In this role, PARP1 binds damaged DNA and this interaction stimulates auto-PARsylation of PARP1 and trans-PARsylation of additional DNA repair mediators such as XRCC1 and chromatin structure proteins such as histones. Ultimately, these PARSylation events orchestrate a molecular cascade that results in the repair of DNA lesions such as single-strand DNA breaks (SSBs; Hottiger et al, 2010).

The demonstration that PARP1 is a critical component of the DNA damage response machinery stimulated the development of small molecule PARP inhibitors as potential therapeutic agents for cancer. The majority of these agents mimic β-NAD^+^ and in doing so inhibit the catalytic activity of PARP enzymes. Clinically, PARP inhibitors could have potential as chemo- and radiosensitizing agents and the demonstration that PARP inhibitors are selective for tumour cells with homologous recombination (HR) gene defects (such as loss-of-function *BRCA1* or *BRCA2* mutations), suggests that these molecules could have some utility as single agents [reviewed in (Lord & Ashworth, 2012)]. For example, Phase I and II clinical trials have shown that the PARP inhibitor olaparib (AZD2281) can elicit significant and sustained anti-tumour responses, especially in familial cancer patients with *BRCA1* or *BRCA2* mutant tumours [reviewed in (Lord & Ashworth, 2012)]. In addition, when used as maintenance therapy after the use of DNA-damaging chemotherapy, olaparib can significantly extend the time to progression of high-grade serous ovarian cancer (Ledermann et al, 2011), a disease where tumours are characterized by a relatively high frequency of HR gene mutations (TCGA, 2011). However, the overall effectiveness of single agent PARP inhibitors in other cancer types has been relatively disappointing (Lord & Ashworth, 2012), although larger studies are required to conclusively assess the performance of PARP inhibitors in diseases such as triple-negative (TN) breast cancer, where tumours are characterized by an absence of estrogen receptor (ER) and progesterone receptor (PR) expression as well as an absence of *HER2* gene amplification (Foulkes et al, 2010). The rationale for targeting TN breast cancer with PARP inhibitors is based upon studies that suggest some level of phenotypic/molecular overlap between TN and *BRCA1* mutant familial breast cancers and the hypothesis that there may be a subset of sporadic breast cancers that could therefore respond favourably to PARP inhibitors (Turner et al, 2004). However, it may well be the case that the optimal use of PARP inhibitors in TN breast cancer might require a combination strategy, either with an existing chemotherapeutic or with a novel targeted agent.

To date, a number of pre-clinical PARP inhibitor combination effects have been reported, notably the synergy between PARP inhibitors and the chemotherapeutic temozolomide (Daniel et al, 2010). In addition, genetic screens have been used to identify candidate combination effects when PARP inhibitors are combined with genetic inhibition of pharmacologically tractable proteins such as kinases (Turner et al, 2008). Each of these efforts has suggested either potential therapeutic combinations or has extended our understanding of the mechanism of action of PARP inhibitors.

We reasoned that as PARP enzymes utilize β-NAD^+^ as a substrate, modifying the activity of other NAD metabolism enzymes might modulate the response to PARP inhibitors. To address this possibility, we performed an RNA interference (RNAi) screen of a panel of genes with a known or proposed role in NAD metabolism to identify new determinants of sensitivity to PARP inhibitor. Given the interest in TN breast cancer and the clear need to identify therapeutic approaches for this disease, we focused these screening efforts on this breast cancer subtype.

## RESULTS

**Figure 1:**
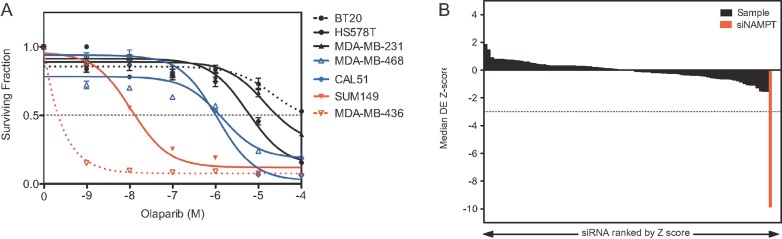
A PARP inhibitor sensitization screen in a TN breast cancer cell line model PARP inhibitor sensitivity in a panel of TN models. Two-week olaparib survival curves from six-well plate clonogenic assays are shown.Rank order of drug effect (DE) Z scores from the PARP inhibitor sensitivity screen. Black bars represent Z scores from sample siRNA SMARTPools targeting 44 protein PARPs and β-NAD^+^-metabolizing genes. The red bar represents the siNAMPT effect, similar in scale to that observed with silencing of *BRCA1* (Turner et al, 2008).

To gain greater insight into extending the utility of PARP inhibitors in the clinic, we assessed the possibility that inhibition of proteins involved in β-NAD^+^ metabolism might modulate the cellular response to a clinical PARP inhibitor, olaparib. To assess this in a relatively unbiased fashion, we conducted a RNAi sensitization screen using olaparib, a potent PARP inhibitor, and a bespoke short-interferring (si)RNA library targeting a panel of 44 genes encoding PARP superfamily members and other proteins involved in β-NAD^+^ metabolism (Supporting Information Table S1). To identify an appropriate TN breast cancer cell line model for screening, we first tested a panel of TN breast cancer cell lines for olaparib sensitivity using a clonogenic assay format, exposing cells to olaparib for 2 weeks (Fig 1A). This confirmed the profound sensitivity of *BRCA1* mutant models (surviving fraction 50 concentration, SF_50_, for SUM149 = 0.01 µM and MDA-MB-436 SF_50_ = 0.0002 µM), the comparative olaparib resistance of TN models such as CAL51, MDA-MB-468, HS578T and MDA-MB-231 (SF_50_ = 1–27 µM) and the more significant resistance of the BT20 TN breast tumour cell line model, which did not reach SF_50_ within the concentration range 1 nM to 100 µM (Fig 1A and Supporting Information Table S3A). So as to maximize the potential for identifying sensitization effects, we selected the relatively insensitive CAL51 TN model for RNAi screening. CAL51 cells were reverse-transfected with siRNA SMARTPools (encompassing four different siRNAs per gene) arrayed in a 96-well-plate format (Turner et al, 2008). Immediately after addition of transfection reagent, we divided cultures into replica plates (Supporting Information Fig S1A). Forty-eight hours after transfection, media containing olaparib was added to half the plates and media containing the drug vehicle, DMSO, added to the other half. To maximize the potential for identifying novel PARP inhibitor sensitization effects, we used a concentration of olaparib that elicited 20% inhibition of cell viability in 96-well-pate format (SF_80_ = 1 µM; Turner et al, 2008). After 5 days continuous culture in the presence of olaparib, cell viability was estimated and comparison of viability data from drug- and vehicle-treated plates used to estimate the effect of siRNA on the cellular response to olaparib (Supporting Information Fig S1A). After data normalization, we classified the effect of each siRNA upon olaparib sensitivity according to a Z score (Boutros et al, 2006; Fig 1B). The most significant effect (Z score −9.87) was caused by a siRNA SMARTPool targeting nicotinamide phosphoribosyltransferase (NAMPT also known as PBEF1—Fig 1B and Supporting Information Table S2). In similar RNAi screens, Z scores of this magnitude had been observed with siRNA targeting the HR gene *BRCA1* (Turner et al, 2008).

**Figure 2:**
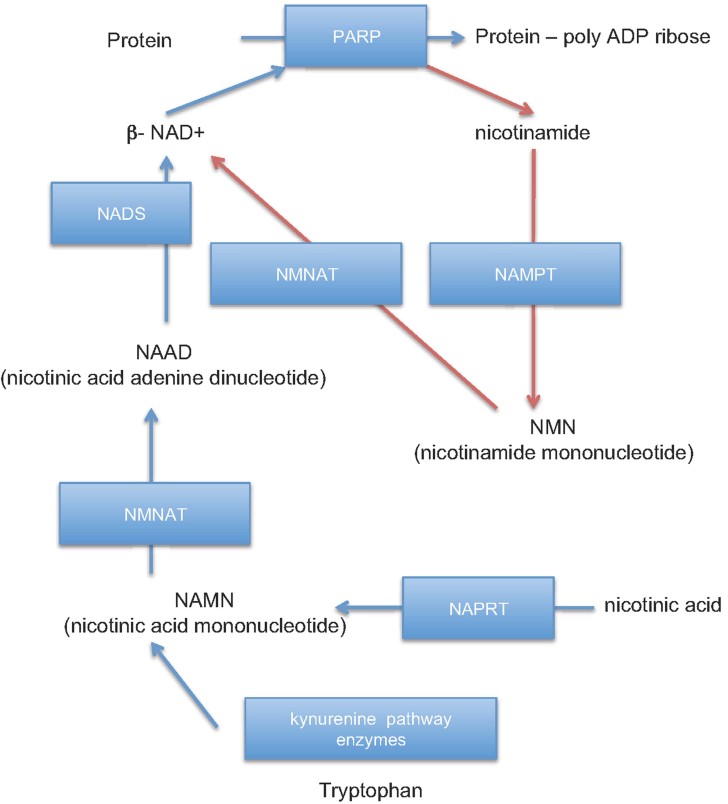
A schematic of mammalian NAD metabolism Metabolites are shown in plain text and enzymes in blue boxes. Abbreviations for enzymes are as follows: PARP, poly(ADP-ribose) polymerase; NAMPT, nicotinamide phosphoribosyl transferase; NMNAT, nicotinamide mononucleotide adenylyl transferase; NAPRT, nicotinic acid phosphoribosyl transferase; NADS, NAD synthase. PARP reactions require β-NAD^+^ as a substrate and generate nicotinamide as a by-product of the PARSylation of proteins. In cells where PARPs are highly active, β-NAD^+^ is largely provided by a salvage pathway that utilizes nicotinamide. Nicotinamide is processed back to β-NAD^+^ by two enzymes, NAMPT and NMNAT, with NAMPT catalysis representing the rate limiting step in this process. Alternatively, β-NAD^+^ can be synthesized *de novo* from either nutritional tryptophan (via the kynurenine pathway) or via NA, which is processed by NAPRT, NMNAT and NADS.

siRNAs targeting NAMPT were included in the bespoke library as NAMPT mediates a rate-limiting step in β-NAD^+^ metabolism. β-NAD^+^, the co-factor used by PARP enzymes as part of the PARsylation reaction, can be biosynthesized in mammalian cells from either nicotinamide (NAM), nicotinic acid (NA) or tryptophan, with NAM being the predominantly used precursor (Garten et al, 2009). The rate-limiting step in β-NAD^+^ synthesis from NAM is the transfer of a phosphoribosyl group onto NAM; this latter reaction is catalysed by NAMPT (Fig 2; Garten et al, 2009).

**Figure 3:**
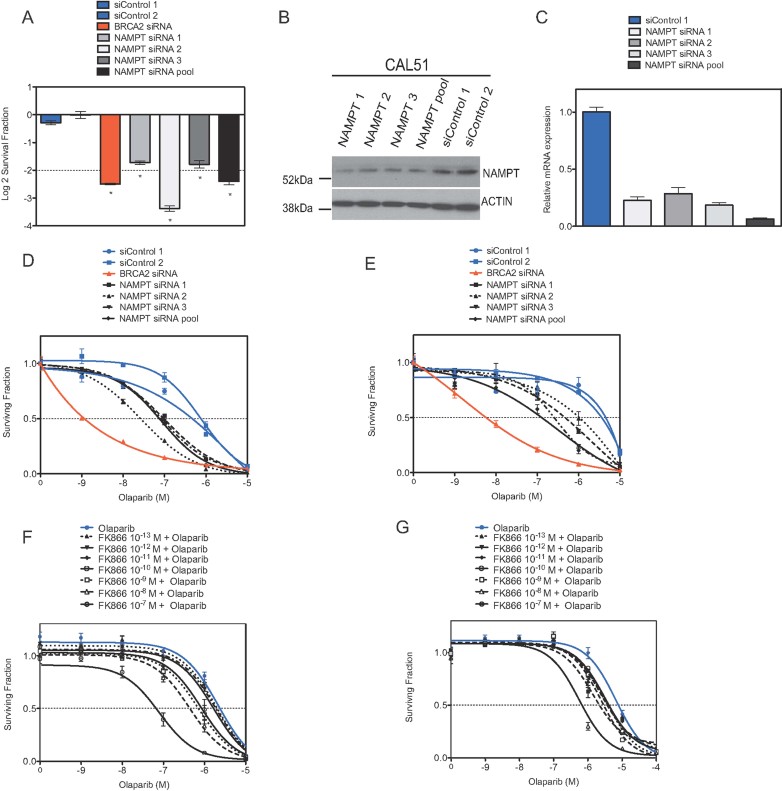
Inhibition of NAMPT sensitizes tumour cells to olaparib **A.** CAL51 cells were transfected with siRNA in a 96-well plate format and then exposed to olaparib (1 µM final concentration) for 5 days after which cell viability was estimated. Log_2_ SFs in 1 µM olaparib (normalized to vehicle-treated cells) are shown. Error bars represent the standard error of the mean (SEM) from three independent experiments; *p-values *versus* siCONTROL1 transfected cells *p* < 0.0001 (Student's *t*-test).**B.** Validation of siRNA silencing effects. CAL51 cells were transfected with siRNA as shown and cell lysates generated 48 h later. Lysates were Western blotted and immunoprobed using an anti-NAMPT (Cell Signalling) antibody as shown.**C.** Validation of siRNA silencing effects using RT-PCR. CAL51 cells were transfected with siRNA as shown and cDNA generated 48 h later. Relative NAMPT mRNA expression (compared to siCONTROL1-transfected cells) is shown.**D,E.** Dose–response sensitization to a PARP inhibitor caused by NAMPT silencing. CAL51 (**D**) or HeLa (**E**) cells were transfected as before and plated in media containing olaparib as shown. Cell viability was determined after 5 days olaparib exposure using Cell Titre Glo. Survival curves are shown. Error bars represent the SEM from three independent experiments. The effects of siRNA targeting *BRCA2* are shown as a positive control; *p*-values for the siNAMPT-transfected cells *versus* siCONTROL1-transfected cells *p* < 0.05 (ANOVA) in CAL51 (**D**) and Hela (**E**). See also Supporting Information Table S3.**F,G.** Inhibition of NAMPT using a potent inhibitor FK866 sensitizes tumour cells to olaparib. CAL51 (**F**) or HeLa (**G**) cells were plated in 96-well plates and exposed to FK866 and/or olaparib, as shown, for 5 days. Cell viability after this time was estimated using Cell Titre Glo (Promega). Survival curves are shown. Error bars represent the SEM from three independent experiments; *p*-values for CAL51 cells exposed to 10^−10^–10^−7^ M FK866 inhibitor and olaparib *versus* olaparib alone *p* < 0.05 (ANOVA), all other comparisons returned non significant *p*-values; *p*-values for HeLa cells exposed to 10^−13^–10^−7^ M FK866 inhibitor and olaparib *versus* olaparib alone *p* < 0.05 (ANOVA). See also Supporting Information Table S3.

RNAi screens, whilst potentially informative, are also prone to off-target effects caused by siRNA reagents (Echeverri et al, 2006). To reduce the possibility that the NAMPT effect identified in the screen was an off-target effect, we repeated the screen assay using three different siRNA species, each targeting NAMPT. Each individual siRNA not only efficiently silenced NAMPT at both the mRNA and protein level but also caused significant olaparib sensitivity (Fig 3A–C). To assess the magnitude of olaparib sensitivity, we used NAMPT siRNA in olaparib dose–response survival experiments, demonstrating that each of the siRNA species sensitized not only CAL51 cells but also a commonly used PARP inhibitor resistant tumour cell line, HeLa (Fig 3D and E). In these experiments, NAMPT siRNA caused as much as a 29-fold increase in olaparib sensitivity (SF_50_ for control (non targeting) siRNA transfected CAL51 cells = 758 nM *vs.* 26 nM for NAMPT siRNA*2 transfected cells, Supporting Information Table S3).

A number of relatively potent NAMPT small molecule chemical inhibitors exist, including FK866, a non-competitive inhibitor of NAMPT that is able to reduce cellular β-NAD^+^ levels (Hasmann & Schemainda, 2003). We assessed the possibility that chemical inhibition of NAMPT could also cause sensitization to olaparib. This proved to be the case; whilst FK866 caused almost negligible cell inhibition when used in the 0.1 pM to 0.1 µM concentration range (Supporting Information Fig S2A and B), it caused a dose-dependent increase in olaparib sensitivity in both CAL51 and HeLa cells (Fig 3F and G and Supporting Information Table S3). In these experiments, FK866 inhibitor caused a 36-fold increase in CAL51 olaparib sensitivity when 0.1 µM FK866 was combined with olaparib and a 12-fold increase in HeLa cells (Supporting Information Table S3).

**Figure 4:**
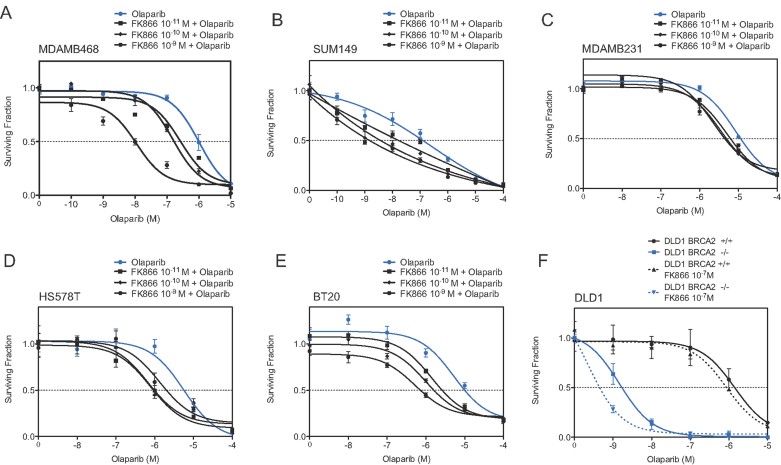
Inhibition of NAMPT using the potent inhibitor FK866 sensitizes additional tumour cells to olaparib **A-E.** Sensitivity of the cell lines MDAMB468 (**A**), SUM149 (**B**), MDAMB231 (**C**), HS578T (**D**) and BT20 (**E**) was assessed as in Fig 3.**F.** Inhibition of NAMPT using the potent inhibitor FK866 sensitizes DLD1 *BRCA2*^−/−^ tumour cells to olaparib. Viability was measured after 14 days of continuous exposure to FK866/olaparib. Error bars represent the SEM from three independent experiments. ANOVA analysis tables are shown in Supporting Information Table S4.

To further assess the generality of our observations, we addressed the possibility that NAMPT inhibition could also increase the therapeutic effect of olaparib in additional TN breast cancer cell lines. FK866 exposure caused clear olaparib sensitivity in four out of five TN models (MDA-MB-468, SUM149, HS578T and BT20 but not MDA-MB-231, Fig 4A–E and Supporting Information Fig S2C–G and Table S4), whilst having negligible effects on the normal breast epithelial line MCF10A (Supporting Information Fig S3A and B). The increased sensitivity of SUM149 (Fig 4B) was notable in that this model is *BRCA1*-deficient, suggesting that FK866 could also increase the sensitivity of HR-null tumour cells to PARP inhibition. To formally test whether the combination of FK866 and olaparib could increase the therapeutic window in a HR-deficient setting, we assessed the effect of FK866/Olaparib on isogenic DLD1 *BRCA2*^+/+^ and *BRCA2*^−/−^ cells. When used as a single agent, 1 nM olaparib caused a 36% inhibition in the surviving fraction (SF) of *BRCA2*^−/−^ cells, while having no effect on *BRCA2*^+/+^ cells (Fig 4F). However, the addition of 0.1 µM FK866 increased this inhibitory effect to 72%, whilst having undetectable effects on *BRCA2*^+/+^ cells [Fig 4F, *BRCA2*^−/−^ 1 nM olaparib *vs. BRCA2*^−/−^ 1 nM olaparib combined with 0.1 µM FK866, *p* < 0.05 (Student's *t*-test)—see also Supporting Information Table S4], an increase in the therapeutic effect also validated by siRNA silencing of NAMPT [Supporting Information Fig S3C, *BRCA2*^−/−^ 1 nM olaparib siCONT transfected cells *vs. BRCA2*^−/−^ 1 nM olaparib siNAMPT transfected cells, *p* < 0.001 (Student's *t*-test)].

**Figure 5:**
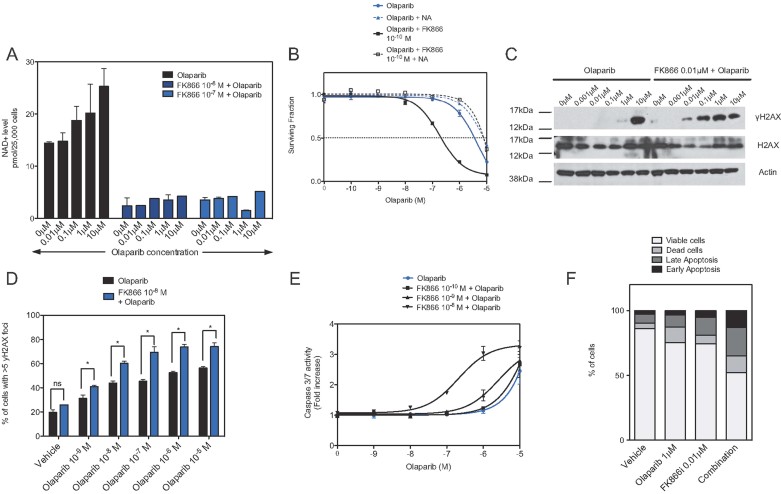
Mechanism of FK866/olaparib synergy Cellular β-NAD^+^ levels in cells exposed to olaparib and/or FK866. CAL51 cells were exposed to FK866 and/or olaparib for 48 h after which β-NAD^+^ levels were estimated using an NAD/NADH Assay Kit (Abcam).Nicotinic acid (NA) rescues the combined effect of olaparib and FK866. CAL51 cells were exposed to olaparib and FK866 as shown in addition to 10 µM NA and cell viability estimated after 6 days drug exposure. Error bars represent the SEM from three independent experiments; *p*-value for CAL51 cells treated with FK866/olaparib *versus* FK866 inhibitor/olaparib plus 10 µM NA, *p* < 0.05 (ANOVA).FK866 exacerbates levels of γH2AX caused by olaparib. CAL51 cells were exposed to FK866 and/or olaparib for 48 h and cell lysates generated and immunoblotted for total and γH2AX.Quantification of γH2AX foci in CAL51 cells. Cells were exposed to FK866 and/or olaparib as in (**C**). Bar chart shows the median number of cells with >5 γH2AX foci per nucleus. Error bars represent three standard deviations of the mean; *p-values for CAL51 cells treated with combination of FK866/olaparib *versus* olaparib alone *p* < 0.05 (Student's *t*-test); ns, not significant *p* > 0.05.Combination of FK866 inhibitor and olaparib causes an increase in apoptosis as measured by caspase 3/7 activity. Cells were treated as in (**C**) and caspase 3/7 activity assessed as in the Materials and Methods section. *p*-values for CAL51 cells treated with 0.01 µM FK866 and olaparib *versus* olaparib alone *p* < 0.05 (ANOVA), all other comparisons returned non-significant *p*-values.Frequency of apoptotic cells in CAL51 cells exposed to FK866 and/or olaparib. Cells were treated as in (**C**) and the frequency of Annexin V-positive cells estimated by FACS analysis.

Given the role of NAMPT in β-NAD^+^ metabolism (Fig 2), we postulated that β-NAD^+^ depletion could be the cause of the synergy observed between FK866 inhibitor and olaparib. To address this possibility, we measured cellular β-NAD^+^ levels using an NAD/NADH cycling assay. As PARP1 consumes β-NAD^+^ as part of its catalytic activity, we expected levels of cellular β-NAD^+^ to increase in response to olaparib exposure. We found that after 48 h olaparib exposure, β-NAD^+^ levels were increased in a concentration dependent manner (Fig 5A, black bars), thus validating the assay system used. We also noted that exposure of cells to FK866 reduced the level of β-NAD^+^ and suppressed the elevation in β-NAD^+^ caused by olaparib exposure (Fig 5A). These observations were consistent with the role of NAMPT in catalysing the rate-limiting step in β-NAD^+^ production (Fig 2). On the basis of these observations, we hypothesized that restoration of β-NAD^+^ levels using artificial supplementation with NA, a substrate for the synthesis of β-NAD^+^ in an NAMPT-independent fashion (Watson et al, 2009; Weidele et al, 2010), might biochemically rescue this effect. To test this, we performed cell survival assays in CAL51 cells exposed to a combination of FK866 and olaparib in the presence or absence of NA. Artificial supplementation with NA rescued CAL51 cells from the combined effect of FK866 and olaparib (Fig 5B), supporting the hypothesis that β-NAD^+^ depletion was in fact the cause of the synergy observed between FK866 inhibitor and olaparib.

In light of these observations, we propose the following scenario to explain the synthetic lethality between PARP and NAMPT inhibition: (i) drugs such as olaparib cause cell inhibition by causing persistent DNA lesions and/or impairing DNA repair; (ii) as olaparib is a reversible catalytic inhibitor that competes with β-NAD^+^ for binding to the catalytic domain of PARP1/2, cellular levels of β-NAD^+^ could, in principle modulate the cell inhibitory effects of olaparib; and (iii) as the major source of β-NAD^+^ for PARsylation reactions is via nicotinamide salvage and the activity of NAMPT, non-competitive inhibition of NAMPT (*e.g.* by the use of an RNAi reagent or a non-reversible catalytic inhibitor such as FK866) could limit β-NAD^+^ levels, reduce the extent of β-NAD^+^/PARP inhibitor competition for the PARP catalytic domain and thus exacerbate the deleterious effects of PARP inhibitors on cells.

To directly assess whether such deleterious effects were in fact exacerbated by FK866, we estimated the extent of potentially lethal DNA lesions caused by PARP inhibitors. PARP inhibitors cause cell inhibition in part by inducing DNA replication fork stalling and double-strand breaks (Farmer et al, 2005). In response to these events, H2AX histones that flank DNA at the damage site are phosphorylated, forming the γH2AX isoform that mark these DNA lesions (Bonner et al, 2008). The estimation of γH2AX, either by Western blotting or immunohistochemistry (*i.e.* the detection of nuclear γH2AX foci), is therefore routinely used to estimate the extent of DNA damage caused by PARP inhibition (Bonner et al, 2008). As expected, H2AX phosphorylation could be detected by Western blotting when cells were exposed to 10 µM olaparib (Fig 5C). Whilst FK866 did not in itself induce H2AX phosphorylation, combining FK866 with olaparib caused detectable γH2AX as measured by Western blotting at concentrations of olaparib as low as 0.01 µM (Fig 5C). Furthermore, the induction of nuclear γH2AX foci was also exacerbated when FK866 was combined with olaparib (Fig 5D and Supporting Information Fig S4A). In some cell types, the DNA damage caused by olaparib induces apoptosis (Farmer et al, 2005). Using a caspase 3/7 activity assay as well as Annexin V immunostaining and subsequent FACS analysis, we noted that the addition of FK866 to olaparib increased the frequency of cells expressing these apoptotic markers when compared to FK866 treatment alone (Fig 5E and F and Supporting Information Fig S4B). Taken together, this data suggested that the combination of FK866 and olaparib most likely enhanced the frequency of potentially lethal DNA lesions caused by PARP inhibitors and in doing so, induces cell death by apoptosis.

**Figure 6:**
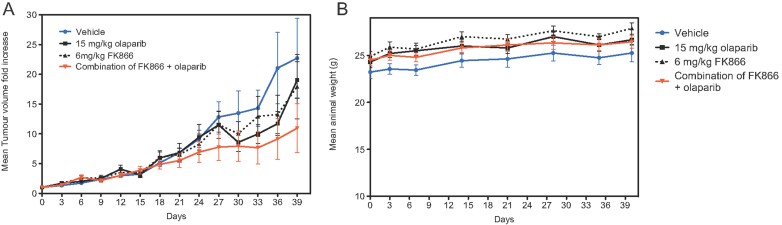
*In vivo* efficacy of olaparib in combination with FK866 inhibitor in TN breast cancer xenografts CAL51 cells were xenografted into immunocompromised mice and once xenografts had established, mice were treated as described in the Materials and Methods section. The therapeutic effect of each therapy compared to vehicle-treated mice is shown for olaparib alone, FK866 alone and the olaparib/FK866 combination. Each data point represents the mean increase in tumour volume after the instigation of treatment and error bars represent SEM, where *n* for each cohort = 10 animals; *p < 0.05 (repeated measures ANOVA with post-test) comparing the FK866/olaparib combination arm *versus* each other cohort, refer to Supporting Information Table S5 for statistical analysis.Body weight in each cohort.

To assess the therapeutic effect of the FK866/olaparib combination *in vivo*, we measured the ability of FK866/olaparib to suppress the progression of a TN cell line-derived xenografted tumour. Triple-negative CAL51 cells were subcutaneously xenografted into female athymic nude mice. Once tumours had established, mice were randomized into one of four treatment groups: (i) a cohort treated with daily olaparib, (ii) a cohort treated with FK866, (iii) a cohort treated with an olaparib/FK866 combination and (iv) a vehicle-treated cohort. Neither single agent olaparib nor FK866 alone suppressed the growth of TN xenografts when compared to the vehicle treatment (Fig 6). However, the combination of FK866 and olaparib caused a clear and statistically significant inhibitory effect on tumour volume, when compared to vehicle treatment or either of the single agent regimes (*p* < 0.05 ANOVA, Fig 6, and Supporting Information Table S5). Most notably, two animals in the combination group (total *n* = 10) showed complete tumour regression by day 39 of treatment with no measurable tumour present at the end of the study, with tumours in the other animals in this cohort exhibiting ostensible disease stabilization. Although xenograft studies such as these are able to generate proof-of-concept data that a human tumour cell can be inhibited *in vivo*, they are relatively limited in their ability to model many forms of clinical toxicity, including those seen in some patients treated with PARP inhibitors such as olaparib. As such it is not clear whether long-term treatment with an NAMPT inhibitor and a PARP inhibitor would lead to deleterious side effects in a clinical setting. Nevertheless, we did note that in the xenograft experiment, each of the treatment regimes was equally well tolerated, with none of the mice showing a significant change in body weight (Fig 6B).

**The paper explained**

PROBLEM:

PARP inhibition represents a promising therapeutic approach for cancer. However, it is not yet clear as to whether single agent PARP inhibitor therapy or combination therapy using these drugs would be most beneficial.

RESULTS:

Here, we show that targeting of the β-NAD^+^ metabolism enzyme NAMPT can increase the tumour cell inhibitory effect of the clinical PARP inhibitor olaparib. NAMPT catalyses a rate limiting step in the generation of the PARP substrate β-NAD^+^, suggesting a likely mechanism of action for these effects. Importantly, inhibition of NAMPT can increase the *in vitro* and *in vivo* effects of olaparib in models of triple-negative breast cancer, a subtype of particular unmet clinical need.

IMPACT:

As both small molecule NAMPT and PARP inhibitors are currently in clinical development, these observations highlight the potential for using combination therapy that involves modulators of β-NAD^+^ metabolism.

## DISCUSSION

One of the goals of pre-clinical and clinical drug development is the identification of evidence-based drug combination strategies that can deliver a therapeutic benefit. Already a number of drug combination strategies involving PARP inhibitors have been proposed based on *in vitro* studies, but in general the majority of these focus on the addition of either DNA damaging agents or additional DNA repair enzyme inhibitors. In the clinical setting, the majority of PARP inhibitor combination trials have focused upon combining PARP inhibitors with standard of care therapies [reviewed in (Lord & Ashworth, 2012)]. Here, we show that by modulating another element of the NAD metabolism network, synthetic lethality/sickness and sensitization to a clinical PARP inhibitor can be achieved. These observations are likely underpinned by the role of NAMPT in modulating β-NAD^+^ levels. We also show, using both genetic and chemical inhibition, that NAMPT inhibition can increase the effect of olaparib in a number of tumour cell models for a disease of particular unmet therapeutic need, TN breast cancer. In addition, the increase in the therapeutic effect of olaparib caused by FK866 in *BRCA2* mutant models, suggests that some level of tumour specificity could be achieved with a NAMPT/PARP inhibitor combination and that genetic mutations that predict loss of HR could provide biomarkers to direct its use. Whilst we have focused upon the effect that NAMPT inhibition can have on DNA damage biomarkers such as nuclear γH2AX foci formation, it is also possible that NAMPT inhibition could also impinge upon other mechanisms that other tumour cells are reliant upon. Whether this is the case or not, it seems clear that NAMPT is a non-redundant modifier of the tumour cell response to a clinical PARP inhibitor; this could imply that elevated levels of NAMPT activity could cause PARP inhibitor resistance, a hypothesis that could be tested in tumour biopsies derived from clinical trials involving single agent PARP inhibitor use. With this possibility in mind, we propose that a further analysis of NAD metabolism enzymes could offer important information in terms of potential combination therapies as well as mechanisms of PARP inhibitor resistance.

## MATERIALS AND METHODS

### Materials

The PARP inhibitor AZD2281/Olaparib (Selleck, Chemical) and NAMPT inhibitor FK866 (Cayman, Chemical) were used as previously described (Farmer et al, 2005; Hasmann & Schemainda, 2003). NA (Sigma–Aldrich) was used as described previously (Watson et al, 2009).

### Cell culture and RNAi screening

All cell lines were obtained from American Type Culture Collection (ATCC) and Horizon Discovery Ltd. and maintained according to the supplier's instructions. Cell lines were transfected with SMARTpool siRNAs, using Lipofectamine 2000 (Invitrogen) transfection reagent. The siRNA library (siARRAY — targeting 44 known and putative human PARPS and proteins with an established role in NAD metabolism) was purchased from Dharmacon. Each well in this library contained a SMARTpool of four distinct siRNA species targeting different sequences of the target transcript. Each plate was supplemented with siCONTROL (10 wells; Dharmacon). RNAi screening conditions were optimized and used as described elsewhere (Lord et al, 2008). Raw Cell Titre Glo luciferase readings from the RNAi screen were processed as described in (Lord et al, 2008). In brief, Cell Titre Go readings were first log_2_ transformed and then centred according to the plate log_2_ transformed median. The effect on PARP inhibitor sensitivity caused by each siRNA was calculated according to the following equation: Drug effect (DE) of siRNA for gene *X* = (median centred data from olaparib-treated wells for gene *X*) − (median centred data from DMSO-treated wells for gene *X*). Calculation of the median absolute deviation (MAD) was used to estimate the variance of the DE data. Standardized Z scores for DE were calculated using DE values, the median DE from the entire library and the MAD as variables. A similar analysis was conducted to estimate the effect of each siRNA upon cell viability in the absence of Olaparib and siRNAs that caused significant cell inhibition alone (*Z* < −3) were excluded from the final analysis. In total, we used data from three biological replicate screens in the final analysis.

### Cell-based assays

Clonogenic survival assays were performed as previously described (Edwards et al, 2008; Farmer et al, 2005). For measurement of sensitivity to PARP inhibitor, exponentially growing cells were seeded in six-well plates at a concentration of 1000–2000 cells per well. For PARP inhibitor, cells were continuously exposed to the drug with media and drug replaced every 72 h. After 15 days, cells were fixed and stained with sulphorhodamine-B (Sigma, St. Louis, USA) and a colorimetric assay performed as described previously (Edwards et al, 2008). SFs were calculated and drug sensitivity curves plotted as previously described (Farmer et al, 2005).

Short-term survival assays were performed in 96-well plates. For measurement of sensitivity to PARP inhibitor and FK866, cells were seeded in 96-well plates at a concentration of 1000–2000 cells per well. Twenty-four hours post-seeding, drug treatment was initiated and cells were continuously exposed to the drug with media and drug replenished 48 h post-initial treatment. After 7 days, cell viability was estimated using Cell-Titre Glo (Promega). SFs were calculated and drug sensitivity curves plotted as previously described (Farmer et al, 2005).

The ApoTox Glo assay (Promega) was used to assess caspase 3/7 activity as per the manufacturer's intructions. CAL51 cells were plated in 96-well plates at the density of 5000 cells per well. Twenty-four hours post-seeding, drug treatment was initiated and maintained for 48 h prior to caspase 3/7 analysis. Annexin V immunostaining and FACS analysis was performed as previously described (Farmer et al, 2005).

### Protein analysis

Whole-cell protein extracts were prepared from cells lysed in NP250 buffer (20 mM Tris pH 7.6, 1 mM EDTA, 0.5% NP40, 250 mM NaCl); supplemented with protease inhibitor cocktail tablets (Roche, Burgess Hill, UK). Protein concentrations were measured using BioRad Protein Assay Reagent (BioRad, Hemel Hempstead, UK). For Western blot analysis, 50 µg of whole cell lysates were electrophoresed on Novex 4–12% gradient *bis*–*tris* pre-cast gels (Invitrogen) and immunoblotted overnight at 4°C with antibodies targeting the following: NAMPT (Cell Signalling), ACTIN (Santa Cruz Biotech), pH2AX (Millipore) and H2AX (Abnova). Incubation with primary antibody was followed by incubation with a horseradish peroxidase-conjugated secondary antibody and chemiluminescent detection of proteins (Amersham Pharmacia, Cardiff, UK).

### Immunohistochemistry

Nuclear γH2AX foci were visualized and quantified by confocal microscopy as previously described (Farmer et al, 2005).

### NAD/NADH assay

For measurement of cellular NAD levels in response to olaparib and FK866, 200 000 cells were plated into six-well plates and 24 h later drug treatment was initiated. Forty-eight hours later cells were processed using the NAD/NADH kit (Abcam) according to manufacturer's instructions.

### Quantitative RT-PCR

Total RNA from CAL51 cell lines was extracted using Trizol (Invitrogen) according to manufacturer's instructions. cDNA was synthesized using Omniscript Reverse Transcriptase System for RT-PCR (Qiagen) with oligo dT as per manufacturer's instructions. Assay-on-demand primer/probe sets were purchased from Applied Biosystems. Real-Time qPCR was performed on the 790DHT Fast Real-Time PCR System (Applied Biosystems). Gene expression was calculated relative to expression of *GAPDH* endogenous control, and adjusted relative to expression in siCONT cDNA. Samples were quantified in triplicate.

### Xenografts

CAL51 cells were mixed 2:1 in matrigel (BD Biosciences) and then injected into bilateral flanks of 6–8 week old female athymic nude mice (seeding density = 2.5 × 10^6^ cells per graft). Once tumours were palpable (>20 mm^3^), mice with similar volume tumours were collated and randomized into four cohorts, (10 animals per cohort, 40 in total). After randomization, drug dosing was initiated. For olaparib, we administered a 15 mg/kg dose to each animal, once daily, a treatment regime known to elicit *BRCA2* selectivity (Farmer et al, 2005). For FK866, we used a treatment regime previously used elsewhere (Drevs et al, 2003), namely 6 mg/kg daily. Drugs were suspended in 2-hydroxypropyl-b-cyclodextrin (HBC) prior to administration (Farmer et al, 2005). Each animal in the study received one daily intraperitoneal drug administration for five consecutive days, followed by 2 days of no treatment, after which the cycle of 5 days treatment, 2 days no treatment was continued until the end of the study. Tumour volumes were measured every 4 days from the initiation of drug dosing and the results expressed as fold increase in tumour volume relative to that at the first drug administration. All *in vivo* modelling was carried out according to regulations set out in the UK Animals (Scientific Procedures) Act 1986 and in line with a UK Home Office approved project licence held by AA.

### Statistical analysis

Data from the siRNA screen was processed as described as in the main text and also in (Lord et al, 2008). Data from comparative groups in the *in vivo* study and the *in vitro* drug combination work was compared using ANOVA (either two way or two way repeated measures, as appropriate) in the Graphpad Prism software package.

## Supplementary material

Detailed facts of importance to specialist readers are published as ”Supporting Information”. Such documents are peer-reviewed, but not copy-edited or typeset. They are made available as submitted by the authors.
