# Supplementary material for: Synthetic lethality of PARP and NAMPT inhibition in triple-negative breast cancer cells
Source: EMBO Mol Med. 2012 Aug 30;4(10):1087–96. doi: 10.1002/emmm.201201250 (PMC3491838; doi:10.1002/emmm.201201250)
Supplement: Supplementary file 1 [file emmm0004-1087-SD1.pdf]

EMBO
Molecular Medicine

Manuscript EMM-2012-01250

Synthetic lethality of PARP and NAMPT inhibition in triple negative breast cancer cells

Ilirjana Bajrami, Asha Kigozi, Antoinette Van Weverwijk, Rachel Brough, Jessica Frankum, Alan Ashworth and Christopher J. Lord

Corresponding authors: Christopher J. Lord and Alan Ashworth, Institute of Cancer Research

Review timeline:

Submission date:	01 February 2012
Editorial Decision:	25 February 2012
Revision received:	22 May 2012
Editorial Decision:	15 June 2012
Revision received:	28 June 2012
Accepted:	10 July 2012

Transaction Report:

(Note: With the exception of the correction of typographical or spelling errors that could be a source of ambiguity, letters and reports are not edited. The original formatting of letters and referee reports may not be reflected in this compilation.)

1st Editorial Decision 25 February 2012

Thank you for the submission of your manuscript to EMBO Molecular Medicine. We have now heard back from the three referees whom we asked to evaluate your manuscript.

As you will see from the enclosed reports, the referees find the study to be of potential interest, however, they also raise a number of concerns specifically regarding the mechanism of action (Ref.1 and Ref.3), but also concerning the efficacy of the drug regimen (Ref.1 and Ref.2).

Given these evaluations, I would like to give you the opportunity to revise your manuscript, with the understanding that the referees' concerns must be fully addressed and that acceptance of the manuscript would entail a second round of review. Please note that it is EMBO Molecular Medicine policy to allow only a single round of revision and that, as acceptance or rejection of the manuscript will depend on another round of review, your responses should be as complete as possible.

Revised manuscripts should be submitted within three months of a request for revision; they will otherwise be treated as new submissions, except under exceptional circumstances in which a short extension is obtained from the editor. Also, the length of the revised manuscript may not exceed 60,000 characters (including spaces) and, including figures, the paper must ultimately fit onto optimally ten pages of the journal. You may consider including any peripheral data (but not methods in their entirety) in the form of Supplementary information.

I look forward to seeing a revised form of your manuscript as soon as possible.

Yours sincerely,

© European Molecular Biology Organization

Editor
EMBO Molecular Medicine

***** Reviewer's comments *****

Referee #1 (Comments on Novelty/Model System):

The in vitro and in vivo data are clear and convincing. Apart from the apparent lack of toxicity of brief treatment in the mouse model (measured by body weight) there is no information to indicate that this will be a tumour-specific treatment.

Referee #1 (Other Remarks):

This is an interesting, novel, clinically relevant and well-conducted piece of research. Inhibition or knockdown of NAMPT in 3 TNBC cell lines plus HeLa cells is shown to enhance the cytotoxic effects of olaparib, and a modest enhancement of activity is demonstrated in a subcutaneous in vivo model of TNBC.

The proposed mechanism is depletion of NAD+ and it is feasible that depleting NAD+ will reduce the dose of olaparib necessary to inhibit PARP activity. However no data is provided to substantiate this mechanism - what effect does NAMPT inhibition or knockdown have on NAD+ levels in the presence and absence of olaparib?

More importantly there is no information about the mechanism by which this combination of treatments induces cell death. This could occur via unrepaired DNA damage (as is the case with BRCA mutated cells) or via modulation of apoptotic pathways.

Furthermore, no indication is provided, either experimental or speculative, that this combination of treatments will be tumour specific. When given as a single agent, long term exposure to olaparib is required, and toxicity has been observed in the clinical studies. Adding an NAD+ depleting agent may well exacerbate this toxicity; the authors do not provide any data to indicate that tumour cells will be more susceptible to NAD+ depletion than replicating normal tissues.

Hence while this is an interesting observation, the authors need to provide some evidence that the proposed combination treatment will have tumour specificity.

Minor point: the in vivo data are not entirely convincing and the selection of 36 days as the single timepoint for analysis is quesionable.

Referee #2 (Other Remarks):

Review Manuscript EMM-2012-01250 entitled

Synthetic lethality between NAMPT and PARP inhibition in triple negative breast cancer cells

This manuscript is well written and describes the synthetic lethality between PARP inhibition and reducing the activity of NAMPT either by siRNA or inhibition, thus reducing supply of β-NAD+ in triple negative cancer cells. There are only few remarks and one major criticism:

1) β and NAD+ should be written containing a hyphen (β-NAD+).

2) "Poly-ADP-ribose" is mostly found in the literature as "poly(ADP-ribose)". The latter one should be used.

3) In the first sentence of the Introduction, the authors describe PARylation as post-translational modification implicated in biological processes ranging from maintenance of genomic stability to Wnt signaling. At least the last point is not covered by the provided citation (Hottiger et al, 2010), and to my knowledge, there is also only one publication providing some speculative evidence for that. This has to be cited.

4) In the Results & Discussion section (page 8), supplementation with nicotinic acid was used to rescue the effect of NAMPT inhibition. In this context I suggest to include a citation that reports the effect of NA supplementation on cell fate after genotoxic stress, which would emphasize the need of β-NAD+ supply for PARP(1) activity and cell survival. The most recent I know of has been published in Biochemical Pharmacology by Weidele et al. in 2010.

5) The use of additional cell lines in the double-treatment experiments as the authors did is one strong point of this paper. But to me it is unclear, why cell lines have been chosen that show either similar (MDA-MB-468) or even an increased sensitivity (SUM149) as CAL51 cells to olaparib treatment alone. It would be more interesting to see if applying the FK866 inhibitor can also sensitize cells that are more resistant to olaparib, i.e. MDA-MB-231. These experiments have to be included in a revised version of the manuscript.

6) This is the major point of criticism:
I wonder if the double-treatment with olaparib and FK866 in the xenograft model really shows synthetic lethality. Using only one time point out of several for statistical evaluation is not a good way to do it. Additionally, to prove that combinatorial treatment is superior to the single treatments, these three data sets have to be compared to each other and not each separate to vehicle. Maybe the treatment has to be extended up to three two to three months in order to see significant changes. Unfortunately, this most interesting part of the manuscript is the one with the weakest evidence for efficacy of the regimen. Thus, this has to be improved to yield publishable results.

I suggest acceptance of the manuscript after major revision.

Referee #3 (Comments on Novelty/Model System):

In the manuscript by Bajrami et al. the authors describe a novel pathway of synthetic lethality between NAMPT and PARP inhibition in clinical relevant in vitro and in vivo models. With some critical exceptions the paper is of good quality and well written (see comments below). In particular, it is interesting to see that pathways of synthetic lethality other than those of PARP inhibition in combination with defects in homologous recombination seem to exist. In principle this reviewer recommends publication of the manuscript provided that the following suggestions are fully taken into account.

Considering the quality of the Journal, it appears to be crucial to this reviewer that these suggestions and additional experiments are fully implemented prior to publication:

Figure 1: Could the authors specify which color code is used in panel A. Panel B can be moved to the SI section.

Figure 3: The figure legend of panel A should be specified. Which black bar corresponds to which siRNA treatment? Appropriate statistical analyses of the data shown in panel D and E need to be performed (two-way ANOVA followed by an appropriate post test)

Figure 4: For panel A, experiments should be repeated using an appropriate concentration range for FK866. With the concentrations used in the current experiments no dose response effect is visible.

Figure 5: These experiments should be repeated with HeLa cells in order to validate the results shown in Fig3E. The concentration of 1E-10 M FK866 is missing for the SUM149 cell line. In order to compare the effects of FK866 between the MDAMB468 and the SUM149 cell lines, cells should be treated with both concentration of FK866. FK899 needs to be changed into FK866 (panel B). Appropriate statistical testing needs to be performed (Two-way ANOVA).

Figure 6: Appropriate statistical testing needs to be performed (Two-way ANOVA followed by an appropriate post test).

Suppl Fig 1B: The data do not seem to be normally distributed, therefore, instead of the Pearson's correlation coefficient (R2), the Spearman's correlation coefficients (r) should be calculated.

Suppl. Fig 3 B: The effect of an FK866 concentration of 1E-9 M is missing.

To this reviewer the mechanism of action of the revealed synthetic lethal effect is far from clear. The authors speculate that this may be related to a reduction/shortage of intracellular NAD+. This hypothesis could be easily tested experimentally. Several assays are available to determine intracellular NAD levels by using enzymatic or HPLC-based methods.

1st Revision - authors' response 22 May 2012

Referee #1 (Comments on Novelty/Model System):

The in vitro and in vivo data are clear and convincing. Apart from the apparent lack of toxicity of brief treatment in the mouse model (measured by body weight) there is no information to indicate that this will be a tumour-specific treatment.

Our response > We thank the reviewer for their comments regarding our manuscript. As the reviewer suggests, the absence of weight loss in the *in vivo* studies, in the face of xenograft tumour growth inhibition, provides some evidence of a tumour-specific effect. To strengthen the evidence supporting this conclusion, we now show additional data that suggests that the combination of a NAMPT inhibitor with a clinical PARP inhibitor can deliver a therapeutic effect in BRCA2 deficient tumour cells when compared to BRCA2 wild type cells (new Fig 4F).

As the reviewer is aware, olaparib is selective for BRCA2 deficient tumour cells. For example, 1 nM olaparib causes a 36% inhibition of DLD1 *BRCA2* $^{-/-}$ tumour cell growth, whilst having no significant effect on isogenic DLD1 *BRCA2*$^{+/+}$ cells (new Fig 4F). We now show that by adding a minimal amount of the NAMPT inhibitor FK866 to olaparib, the level of *BRCA2* $^{-/-}$ cell growth inhibition can be increased to 72%, with no inhibitory effect on *BRCA2*$^{+/+}$ cells (new Fig 4F). We also now show that the therapeutic effect of olaparib can be enhanced in DLD1 *BRCA2* $^{-/-}$ cells when NAMPT gene expression is inhibited by RNA interference (new Fig S3C of Supporting Information). Given the tumour specificity of *BRCA2* mutations, this new data suggests that the NAMPT inhibitor/PARP inhibitor combination could deliver a profound tumour specific effect.

In addition to using the DLD1 isogenic cell line to illustrate tumour specificity we have also now assessed the effects of the FK866/olaparib combination on MCF10A, a non-tumorigenic breast epithelial cell line; here the combination does not cause any increase in sensitivity in this normal epithelial cell line model (new Fig S3A and B of Supporting Information), suggesting, to some extent, a minimal effect on normal cells. Taken together with the *in vivo* experiments described in the paper, it seems reasonable to us to propose that there might be some benefit in the combination of NAMPT inhibition and a clinical PARP inhibitor.

Referee #1 (Other Remarks):

© European Molecular Biology Organization

This is an interesting, novel, clinically relevant and well-conducted piece of research. Inhibition or knockdown of NAMPT in 3 TNBC cell lines plus HeLa cells is shown to enhance the cytotoxic effects of olaparib, and a modest enhancement of activity is demonstrated in a subcutaneous in vivo model of TNBC.

The proposed mechanism is depletion of NAD+ and it is feasible that depleting NAD+ will reduce the dose of olaparib necessary to inhibit PARP activity. However no data is provided to substantiate this mechanism - what effect does NAMPT inhibition or knockdown have on NAD+ levels in the presence and absence of olaparib ?

Our response > Given the role of NAMPT (Fig 2), we postulated that b-NAD^+ depletion could be the cause of the synergy observed between FK866 inhibitor and olaparib. To address this issue, we have now estimated cellular b-NAD^+ levels using an NAD/NADH cycling assay (new Fig 5A).

As PARP1 consumes b-NAD^+ as part of its catalytic activity, we expected levels of cellular b-NAD^+ to increase in response to olaparib exposure, at least in the short term. We found that after 48 hours olaparib exposure, b-NAD^+ levels were increased in a concentration dependent manner (new Fig 5A – black bars), thus validating the assay system used. We also noted that exposure of cells to 0.1 mM or 0.01 mM FK866 reduced the level of b-NAD^+ and suppressed the elevation in b-NAD^+ caused by olaparib. These observations were consistent with the role of NAMPT in catalysing the rate-limiting step in b-NAD^+ production and in concert with the experiments using nicotinic acid to rescue FK866/olaparib synergy (Fig 5B) suggest that b-NAD^+ reduction could underlie the effects we have observed.

Referee #1 > More importantly there is no information about the mechanism by which this combination of treatments induces cell death. This could occur via unrepaired DNA damage (as is the case with BRCA mutated cells) or via modulation of apoptotic pathways.

Our response > To address these issues, we have now assessed a number of mechanistic markers that could help explain the effects we observe. These include a measurement of markers of apoptosis (new Fig 5E,F and Fig S4B of Supporting Information) in addition to estimating the prevalence of double strand breaks/stalled replication forks via the immunodetection of gH2AX both by western blot and confocal microscopy (new Fig 5C,D and Fig S4A of Supporting Information).

These results demonstrate the following: (i) the combination of FK866 and olaparib elicits H2AX phosphorylation and the formation of nuclear gH2AX foci at lower concentrations than for olaparib or FK866 alone (new Fig 5C,D); (ii) the combination of FK866 and olaparib increases caspase 3/7 activity (a surrogate marker of apoptosis) to a greater extent than olaparib alone (new Fig 5E); and (iii) FACS analysis of annexin V stained cells suggests that the proportion of apoptotic cells is

© European Molecular Biology Organization

increased in cells exposed to the combination of FK866 and olaparib to a greater extent than for olaparib alone (new Fig 5F). In light of these observations, we propose the following scenario to explain the synthetic lethality between PARP and NAMPT inhibition: (i) drugs such as olaparib cause cell inhibition by causing persistent DNA lesions and/or impairing DNA repair, (ii) as olaparib is a reversible catalytic inhibitor that competes with β-NAD$^+$ for binding to the catalytic domain of PARP1/2, cellular levels of β-NAD$^+$ could, in principle, modulate the cell inhibitory effects of olaparib, and (iii) as the major source of β-NAD$^+$ for PARsylation reactions is via nicotinamide salvage and the activity of NAMPT, non-competitive inhibition of NAMPT (for example by the use of an RNAi reagent or a non-reversible catalytic inhibitor such as FK866) could limit β-NAD$^+$ levels, reduce the extent of β-NAD$^+$/PARP inhibitor competition for the PARP catalytic domain and thus exacerbate the deleterious effects of PARP inhibitors on cells of drugs such as olaparib.

Referee #1> Furthermore, no indication is provided, either experimental or speculative, that this combination of treatments will be tumour specific. When given as a single agent, long term exposure to olaparib is required, and toxicity has been observed in the clinical studies. Adding an NAD+ depleting agent may well exacerbate this toxicity; the authors do not provide any data to indicate that tumour cells will be more susceptible to NAD+ depletion than replicating normal tissues.

Our response > The reviewer makes a valid point. However it is still not clear whether the toxicities observed in clinical trials using olaparib are due to the effect that olaparib has on NAD metabolism. It is also not clear as to whether the toxicities observed are directly attributable to olaparib treatment or are a secondary effect of prior treatments (such as carboplatin in ovarian cancer) that could significantly deplete lymphocyte numbers and lead to myelosuppression once a patient enters an olaparib trial. Although these issues are almost impossible to address with *in vitro* systems or animal xenograft studies, with our new data on BRCA2 selectivity we have tried to model the comparative effects on the drug combination in tumour and normal cells (see earlier comments). This new data suggests that when used at very low concentrations, the FK866/olaparib combination can deliver a therapeutic effect. Nevertheless, we appreciate the important point that the reviewer has raised and we have now added text to the manuscript that discusses this issue, as follows:

"Although xenograft studies such as these are able to generate proof of concept data that a human tumour cell can be inhibited *in vivo*, they are relatively limited in their ability to model many forms of clinical toxicity, including those seen in some patients treated with PARP inhibitors such as olaparib. As such it is not clear whether long-term treatment with a NAMPT inhibitor and a PARP inhibitor would lead to deleterious side effects in a clinical setting. Nevertheless, we did note that in the xenograft experiment, each of the treatment regimes was equally well tolerated, with none of the mice showing a significant change in body weight (Fig 6D). "

© European Molecular Biology Organization

Referee #1> Hence while this is an interesting observation, the authors need to provide some evidence that the proposed combination treatment will have tumour specificity.

Our response > In addition to the *in vivo* study we have now included Figure 4F and Fig S3C, which show that the therapeutic effect of olaparib can be enhanced in *BRCA2* $^{-/-}$ cells with the addition of NAMPT inhibition (see previous comments).

Referee #1> Minor point: the in vivo data are not entirely convincing and the selection of 36 days as the single timepoint for analysis is quesionable.

Our response > As the reviewer is aware, all of the *in vivo* model systems have significant limitations and here we have selected a xenograft system where we can administer a drug to a human tumour, albeit within a mouse host, simply with the intention of demonstrating that *in vivo*, a relatively PARP inhibitor refractory model, CAL51, can be better inhibited when FK866 is added to olaparib. In our experience, the only way to effectively ablate human xenografts with PARP inhibitors is to initiate olaparib treatment exactly at the same time as the introduction of the xenograft. Whilst this latter approach can in certain circumstances deliver a "curative" effect it probably more likely models a prophylactic treatment rather than the treatment of an established disease. Nevertheless, we understand the referee's concerns and have modified the text, now describing the limitiations of *in vivo* studies to recognise that we are not seeing a curative effect but simply something akin to disease stabilisation (as follows);

"Most notably, two animals in the combination group (total n= 10) showed complete tumour regression by day 39 of treatment with no measurable tumour present at the end of the study, with tumours in the other animals in this cohort exhibiting ostensible disease stabilisation."

In regard to the statistic used to describe the effect, we have now modified the figure so that now we describe the difference between the groups using ANOVA. We have now included Supporting Table 5 to describe the statistical analysis of the *in vivo* study. In brief, the use of ANOVA suggests that the tumour volumes are significantly different at $p<0.05$ for the following comparisons: (i) olaparib/FK866 combination *vs.* vehicle, (ii) olaparib/FK866 combination *vs.* FK866, (iii) olaparib/FK866 combination *vs.* olaparib. As expected for an olaparib refractory xenograft such as CAL51 the following comparisons were not statistically significant: (i) olaparib *vs.* vehicle, (ii) olaparib *vs.* FK866 and (iii) FK866 *vs.* vehicle.

Referee #2:

This manuscript is well written and describes the synthetic lethality between PARP inhibition and reducing the activity of NAMPT either by siRNA or inhibition, thus reducing supply of NAD+ in triple negative cancer cells. There are only few remarks and one major criticism:

1) b-NAD+; and NAD+ should be written containing a hyphen (b-NAD+).

2) "Poly-ADP-ribose" is mostly found in the literature as "poly(ADP-ribose)". The latter one should be used.

Our response > We thank the reviewer for pointing out these errors and these have now been corrected in the text.

Referee #2> 3) In the first sentence of the Introduction, the authors describe PARylation as post-translational modification implicated in biological processes ranging from maintenance of genomic stability to Wnt signaling. At least the last point is not covered by the provided citation (Hottiger et al, 2010), and to my knowledge, there is also only one publication providing some speculative evidence for that. This has to be cited.

Our response > We have now modified the text (and citations) to account for these points, for example:

"The addition of poly(ADP-ribose) chains onto proteins (PARsylation) is a post-translational modification that has been implicated in a wide range of biological processes as diverse as the maintenance of genomic stability (Hottiger et al, 2010) and Wnt signalling (Callow et al, 2011; Huang et al, 2009; Zhang et al, 2011)."

Callow MG, Tran H, Phu L, Lau T, Lee J, Sandoval WN, Liu PS, Bheddah S, Tao J, Lill JR, Hongo JA, Davis D, Kirkpatrick DS, Polakis P, Costa M (2011) Ubiquitin ligase RNF146 regulates tankyrase and Axin to promote Wnt signaling. *PLoS One* **6:** e22595

Huang SM, Mishina YM, Liu S, Cheung A, Stegmeier F, Michaud GA, Charlat O, Wiellette E, Zhang Y, Wiessner S, Hild M, Shi X, Wilson CJ, Mickanin C, Myer V, Fazal A, Tomlinson R, Serluca F, Shao W, Cheng H, Shultz M, Rau C, Schirle M, Schlegl J, Ghidelli S, Fawell S, Lu C, Curtis D, Kirschner MW, Lengauer C, Finan PM, Tallarico JA, Bouwmeester T, Porter JA, Bauer A, Cong F (2009) Tankyrase inhibition stabilizes axin and antagonizes Wnt signalling. *Nature* **461:** 614-620

Zhang Y, Liu S, Mickanin C, Feng Y, Charlat O, Michaud GA, Schirle M, Shi X, Hild M, Bauer A, Myer VE, Finan PM, Porter JA, Huang SM, Cong F (2011) RNF146 is a poly(ADP-ribose)-directed E3 ligase that regulates axin degradation and Wnt signalling. *Nat Cell Biol* **13:** 623-629

Referee #2> 4) In the Results & Discussion section (page 8), supplementation with nicotinic acid was used to rescue the effect of NAMPT inhibition. In this context I suggest to include a citation that reports the effect of NA supplementation on cell fate after genotoxic stress, which would emphasize

the need of NAD+ supply for PARP(1) activity and cell survival. The most recent I know of has been published in Biochemical Pharmacology by Weidele et al. in 2010.

Our response > We have now modified the text accordingly:

"On the basis of these observations, we hypothesised that restoration of β-NAD$^+$ levels using artificial supplementation with nicotinic acid (NA), a substrate for the synthesis of β-NAD$^+$ in an NAMPT independent fashion (Watson et al, 2009; Weidele et al, 2010) might biochemically rescue this effect."

Watson M, Roulston A, Belec L, Billot X, Marcellus R, Bedard D, Bernier C, Branchaud S, Chan H, Dairi K, Gilbert K, Goulet D, Gratton MO, Isakau H, Jang A, Khadir A, Koch E, Lavoie M, Lawless M, Nguyen M, Paquette D, Turcotte E, Berger A, Mitchell M, Shore GC, Beauparlant P (2009) The small molecule GMX1778 is a potent inhibitor of NAD+ biosynthesis: strategy for enhanced therapy in nicotinic acid phosphoribosyltransferase 1-deficient tumors. *Mol Cell Biol* **29:** 5872-5888

Weidele K, Kunzmann A, Schmitz M, Beneke S, Burkle A (2010) Ex vivo supplementation with nicotinic acid enhances cellular poly(ADP-ribosyl)ation and improves cell viability in human peripheral blood mononuclear cells. *Biochem Pharmacol* **80:** 1103-1112

Referee #2> 5) The use of additional cell lines in the double-treatment experiments as the authors did is one strong point of this paper. But to me it is unclear, why cell lines have been chosen that show either similar (MDA-MB-468) or even an increased sensitivity (SUM149) as CAL51 cells to olaparib treatment alone. It would be more interesting to see if applying the FK866 inhibitor can also sensitize cells that are more resistant to olaparib, i.e. MDA-MB-231. These experiments have to be included in a revised version of the manuscript.

Our response > We now include data showing the effect of FK866 and olaparib on other resistant triple negative cell lines; MDAMB231, HS578T and BT20 (the latter line being the most olaparib resistant - new Fig 4C-E and Table S4 of Supporting Information). This new data suggests that FK866 can clearly sensitise HS578T and BT20 cells to olaparib and even causes a relatively modest level of sensitivity in MDA-MB-231 cells.

Referee #2> 6) This is the major point of criticism:

I wonder if the double-treatment with olaparib and FK866 in the xenograft model really shows synthetic lethality. Using only one time point out of several for statistical evaluation is not a good way to do it. Additionally, to prove that combinatorial treatment is superior to the single treatments, these three data sets have to be compared to each other and not each separate to vehicle. Maybe the

treatment has to be extended up to three two to three months in order to see significant changes. Unfortunately, this most interesting part of the manuscript is the one with the weakest evidence for efficacy of the regimen. Thus, this has to be improved to yield publishable results.

Our response > In order to better assess the combinatorial effect of FK866 and olaparib *in vivo* we have now analysed the comparative responses in each group by ANOVA (new Table S5 of Supporting Information – see next page). This analysis suggests that the combinatorial therapy is significantly (p<0.05) more effective in restricting the progression of the xenograft than the vehicle treatment or either of the single agent treatments. Ideally we would like to perform studies where daily treatment over a three-month period can occur but this is not a procedure covered by our Home Office licence. Whilst we have enquired about amending our licence to cover such an experiment, the view of our Ethics Committee is that as statistical significance has been reached in the current study, extending the length of treatment would not be permitted.

I suggest acceptance of the manuscript after major revision.

Referee #3 (Comments on Novelty/Model System):

In the manuscript by Bajrami et al. the authors describe a novel pathway of synthetic lethality between NAMPT and PARP inhibition in clinical relevant in vitro and in vivo models. With some critical exceptions the paper is of good quality and well written (see comments below). In particular, it is interesting to see that pathways of synthetic lethality other than those of PARP inhibition in combination with defects in homologous recombination seem to exist. In principle this reviewer recommends publication of the manuscript provided that the following suggestions are fully taken into account. Considering the quality of the Journal, it appears to be crucial to this reviewer that these suggestions and additional experiments are fully implemented prior to publication:

Figure 1: Could the authors specify which color code is used in panel A. Panel B can be moved to the SI section.

Our response > The colour code in Fig 1A refers to the cell line identity key shown on the right hand side of the figure. We have now moved Panel B to the Supporting information as suggested.

Referee #3> Figure 3: The figure legend of panel A should be specified. Which black bar corresponds to which siRNA treatment? Appropriate statistical analyses of the data shown in panel D and E need to be performed (two-way ANOVA followed by an appropriate post test)

Our response > We have now revised Fig 3 so that the identity of each siRNA in the bar charts is clear. We have also now analysed the data in D and E by ANOVA as suggested. *p* values for the siRNA targeting NAMPT-transfected cells *vs.* siCONTROL1 transfected cells, p<0.05 (ANOVA) in

both cell lines (revised Fig 3 and Table S3 of Supporting Information shows the results of ANOVA for each siRNA targeting NAMPT *vs*. siCONTROL1).

Referee #3> Figure 4: For panel A, experiments should be repeated using an appropriate concentration range for FK866. With the concentrations used in the current experiments no dose response effect is visible.

Our response > We have now carried out the experiment using lower concentrations of FK866 in CAL51 and HeLa cells (new Fig 3F,G and Fig S2A, B and Table S3 of Supporting Information). In CAL51 cells, whilst 0.1 *p*M -10 *p*M FK866 did not increase sensitivity to olaparib, 0.1 *n*M -100 *n*M did. In HeLa cells, 0.1 *p*M FK866 did not significantly increase sensitivity to olaparib, but FK866 used in the range 1 *p*M -10 *n*M caused a modest increase in sensitivity, with the most profound olaparib sensitizing effect being caused by 100 *n*M of FK866.

Referee #3> Figure 5: These experiments should be repeated with HeLa cells in order to validate the results shown in Fig3E. The concentration of 1E-10 M FK866 is missing for the SUM149 cell line. In order to compare the effects of FK866 between the MDAMB468 and the SUM149 cell lines, cells should be treated with both concentration of FK866. FK899 needs to be changed into FK866 (panel B). Appropriate statistical testing needs to be performed (Two-way ANOVA).

Our response > We have now made the suggested changes. These are as follows:

(i) "*experiments should be repeated with HeLa cells in order to validate the results shown in Fig3E" -* New Fig 3G indicates that in HeLa cells sensitisation to olaparib can be caused by FK866.

(ii) "*The concentration of 1E-10 M FK866 is missing for the SUM149 cell line." -* New Fig 4B now includes the data for 1E-10 M FK866 in the SUM149 cell line.

(iii) "*In order to compare the effects of FK866 between the MDAMB468 and the SUM149 cell lines, cells should be treated with both concentration of FK866" -* New Fig 4A,B now includes the addition of further concentrations of FK866 to MDAMB468 and SUM149 cells as suggested.

(iv) "*FK899 needs to be changed into FK866 (panel B)"* – this has now been corrected in the text.

(v) "*Appropriate statistical testing needs to be performed (Two-way ANOVA)"* –we have now included new Supporting Table S3 and S4 which details the two-way ANOVA analysis for the FK866/Olaparib combination experiments.

Referee #3> Figure 6: Appropriate statistical testing needs to be performed (Two-way ANOVA followed by an appropriate post test).

Our response > We have now included Supporting Table S5 to describe the statistical analysis of the *in vivo* study. In brief, the use of ANOVA suggests that the tumour volumes are significantly different at $p<0.05$ for the following comparisons: (i) olaparib/FK866 combination *vs.* vehicle, (ii)

olaparib/FK866 combination *vs.* FK866, (iii) olaparib/FK866 combination *vs.* olaparib. As expected for an olaparib refractory xenograft such as CAL51 the following comparisons were not statistically significant: (i) olaparib *vs.* vehicle, (ii) olaparib *vs.* FK866 and (iii) FK866 *vs.* vehicle.

Table S5 of Supporting Information
In vivo efficacy of olaparib in combination with FK866 inhibitor in triple negative breast cancer xenografts. Repeated measures ANOVA, *p* values of each comparison made in the *in vivo* study.

Referee #3> Suppl Fig 1B: The data do not seem to be normally distributed, therefore, instead of the Pearson's correlation coefficient (R2), the Spearman's correlation coefficients (r) should be calculated.

Our response > As suggested, we have now added the r values, rather than r^2.

Referee #3> Suppl. Fig 3 B: The effect of an FK866 concentration of 1E-9 M is missing.

Our response > We have now modified the figure accordingly (new Fig S2D of Supporting Information).

Referee #3> To this reviewer the mechanism of action of the revealed synthetic lethal effect is far from clear. The authors speculate that this may be related to a reduction/shortage of intracellular NAD+. This hypothesis could be easily tested experimentally. Several assays are available to determine intracellular NAD levels by using enzymatic or HPLC-based methods.

Our response > Given the role of NAMPT (Fig 2), we postulated that b-NAD^+ depletion could be the cause of the synergy observed between FK866 inhibitor and olaparib. To address this issue, we have now estimated cellular b-NAD^+ levels using an NAD/NADH cycling assay (new Fig 5A).

As PARP1 consumes b-NAD^+ as part of its catalytic activity, we expected levels of cellular b-NAD^+ to increase in response to olaparib exposure, at least in the short term. We found that after 48 hours olaparib exposure, b-NAD^+ levels were increased in a concentration dependent manner (new Fig 5A – black bars), thus validating the assay system used. We also noted that exposure of cells to 0.1 mM or 0.01 mM FK866 reduced the level of b-NAD^+ and suppressed the elevation in b-NAD^+ caused by olaparib. These observations were consistent with the role of NAMPT in catalysing the rate-limiting step in b-NAD^+ production and in concert with the experiments using nicotinic acid to rescue FK866/olaparib synergy (Fig 5B) suggest that b-NAD^+ reduction could underlie the effects we have observed.

In addition, we have now assessed a number of other mechanistic markers that could help explain the effects we observe. These include a measurement of markers of apoptosis (new Fig 5E,F and Fig

© European Molecular Biology Organization

S4B of Supporting Information) in addition to estimating the prevalence of double strand breaks/stalled replication forks via the immunodetection of gH2AX both by western blot and confocal microscopy (new Fig 5C,D and Fig S4A of Supporting Information).

These results demonstrate the following: (i) the combination of FK866 and olaparib elicits H2AX phosphorylation and the formation of nuclear gH2AX foci at lower concentrations than for olaparib or FK866 alone (new Fig 5C,D); (ii) the combination of FK866 and olaparib increases caspase 3/7 activity (a surrogate marker of apoptosis) to a greater extent than olaparib alone (new Fig 5E); and (iii) FACS analysis of annexin V stained cells suggests that the proportion of apoptotic cells is increased in cells exposed to the combination of FK866 and olaparib to a greater extent than for olaparib alone (new Fig 5F). In light of these observations, we propose the following scenario to explain the synthetic lethality between PARP and NAMPT inhibition: (i) drugs such as olaparib cause cell inhibition by causing persistent DNA lesions and/or impairing DNA repair, (ii) as olaparib is a reversible catalytic inhibitor that competes with β-NAD^+ for binding to the catalytic domain of PARP1/2, cellular levels of β-NAD^+ could, in principle, modulate the cell inhibitory effects of olaparib, and (iii) as the major source of β-NAD^+ for PARsylation reactions is via nicotinamide salvage and the activity of NAMPT, non-competitive inhibition of NAMPT (for example by the use of an RNAi reagent or a non-reversible catalytic inhibitor such as FK866) could limit β-NAD^+ levels, reduce the extent of β-NAD^+/PARP inhibitor competition for the PARP catalytic domain and thus exacerbate the deleterious effects of PARP inhibitors on cells of drugs such as olaparib.

2nd Editorial Decision 15 June 2012

Thank you for the submission of your revised manuscript to EMBO Molecular Medicine. We have now received the enclosed reports from the referees that were asked to re-assess it. As you will see the reviewers are now globally supportive and I am pleased to inform you that we might be able to accept your manuscript pending the following final amendments and the satisfaction of Referee #2:

As you can see Referee #2 is not entirely satisfied with the responses to reviewers and is still concerned with some issues that I believe should be addressed in full.

On a more editorial matter, few concerns remain that should be adressed too:
- Please make your figures in a portrait format and generate them in a large enough size (and high resolution) so that even if reduced, all labels will remain readable.
- Please provide up to 5 keywords
- In the material and methods, please provide an ethical statement concerning the use of small animals.

Please submit your revised manuscript within two weeks. I look forward to seeing a revised form of your article as soon as possible.

Yours sincerely,

Editor
EMBO Molecular Medicine

© European Molecular Biology Organization

***** Reviewer's comments *****

Referee #1 (Comments on Novelty/Model System):

Comments adequately addressed

Referee #1 (Other Remarks):

The comments made in the previous review have been adequately addressed.

One minor point noted on re-reading the article: page 4 line 5 should read 'significantly extend the time to progression' (not 'reduce the time to progression')

Referee #2 (Other Remarks):

Review Manuscript EMM-2012-01250-V2 entitled

Synthetic lethality between NAMPT and PARP inhibition in
triple negative breast cancer cells

Most of my criticisms have been addressed properly, but nevertheless, the most problematic part about in vivo synthetic lethality has not been solved adequately.

Minor comments:

Authors should use either "Supplementary" or "Supporting", not both.
Statistics in Supplementary Figure 2 are missing.

Major points:

For final acceptance, authors should improve Fig. 5 and respective analyses:

1) Fig. 5A/B/C should be combined in one single panel, similar to the data presented in Fig. 5D.
2) I tried to merge the data into one single panel, and looking at the result I am not convinced that there is a significant difference at day 36, maybe there is one at day 39. In order to clarify this, the original data regarding "median tumor volume, fold increase" should be presented. Or as an alternative, the calculated SEM values can be included in Supplementary Table 5, so everyone can better compare the respective data.
3) What has been compared by ANOVA to yield the p-values in Supplementary Table 5? Is it still day36 alone?
What was been compared? Olaparib/FK866 vs. Olaparib vs. FK866 vs. Vehicle? Or other combinations? This is not clear from the legend.
These modifications are mandatory for publication and I would like to review the revised version before acceptance.

Referee #3 (Other Remarks):

All points have been addressed adequately. I suggest publication without hesitation.

2nd Revision - authors' response 28 June 2012

Referee #1 (Comments on Novelty/Model System):

Comments adequately addressed

Referee #1 (Other Remarks):

The comments made in the previous review have been adequately addressed.

One minor point noted on re-reading the article: page 4 line 5 should read 'significantly extend the time to progression' (not 'reduce the time to progression')

Our response > We have now made this change in the main text. This sentence now correctly reads:

"In addition, when used as maintenance therapy after the use of DNA damaging chemotherapy, olaparib can significantly extend the time to progression of high grade serous ovarian cancer (Ledermann et al, 2011), a disease where tumours are characterised by a relatively high frequency of HR gene mutations (TCGA, 2011)."

Referee #2 (Other Remarks):

Most of my criticisms have been addressed properly, but nevertheless, the most problematic part about in vivo synthetic lethality has not been solved adequately.

Minor comments:

Authors should use either "Supplementary" or "Supporting", not both.

Our response > We thank the reviewer for pointing out this error and have now corrected this in the text of the manuscript.

Statistics in Supplementary Figure 2 are missing.

Our response > Supporting Figure 2 shows the effect of FK866 on cell inhibition We have now added a new Supporting Table (Supporting Table 6) showing Student's *t* test *p* values of each FK866 response compared to the response to vehicle.

Major points:
For final acceptance, authors should improve Fig. 5 and respective analyses:
1) Fig. 5A/B/C should be combined in one single panel, similar to the data presented in Fig. 5D.

Our response > We presume this comment is in reference to Figure 6 that describes the *in vivo* data, rather than Figure 5. We have now combined the panels as suggested in a revised version of Figure 6.

2) I tried to merge the data into one single panel, and looking at the result I am not convinced that there is a significant difference at day 36, maybe there is one at day 39. In order to clarify this, the original data regarding "median tumor volume, fold increase" should be presented. Or as an alternative, the calculated SEM values can be included in Supplementary Table 5, so everyone can better compare the respective data.

3) What has been compared by ANOVA to yield the p-values in Supplementary Table 5? Is it still day36 alone?
What was been compared? Olaparib/FK866 vs. Olaparib vs. FK866 vs. Vehicle? Or other combinations? This is not clear from the legend.

Our response > The referee will have noticed that in the revised version of the manuscript, we have compared the mean tumour volumes in the different treatment cohorts using a repeated measures ANOVA (as described in the Methods section as well as in the legend to Supporting Table 5). This approach takes into account the tumour volumes in each mouse and in each cohort across all time points, rather than a single time point analysis. In the legend to the figure we also stated: "*$p<0.05$ (ANOVA) comparing the FK866/olaparib combination arm *vs.* each other cohort, refer to Table S5 of Supporting information for statistical analysis" to suggest that the mean tumour volume response

© European Molecular Biology Organization

in the FK866/olaparib combination cohort is statistically different than the response in all of the other treatment cohorts (i.e different to the response to in the olaparib single agent cohort, different to the response in the FK866 single agent cohort and different to the response in the vehicle treated cohort).

In Table S5 of Supporting information, the comparisons made also seem clear (for example "Olaparib/FK866 combination vs Olaparib".

Nevertheless, we are happy to revise Figure 6 as the referee suggests (we originally separated the different cohort data for ease of viewing) and add the mean volume data and SEM to Supporting Table S5. Although repeated measures ANOVA analysis in xenograft studies such as the one described here is commonly used, we have also now expanded the legend to Supporting Table 5 to state:

"Table S5A of Supporting Information – page 6
In vivo efficacy of olaparib in combination with FK866 inhibitor in triple negative breast cancer xenografts. Repeated measures ANOVA *p* values of each comparison made in the *in vivo* study using a Newman-Keuls post test. Here, a repeated measures ANOVA analysis was used to compare tumour volumes in different cohorts across all time points."

These modifications are mandatory for publication and I would like to review the revised version before acceptance.

Referee #3 (Other Remarks):

All points have been addressed adequately. I suggest publication without hesitation.
