# Supplementary material for: Synthetic lethality of PARP and NAMPT inhibition in triple-negative breast cancer cells
Source: EMBO Mol Med. 2012 Aug 30;4(10):1087–96. doi: 10.1002/emmm.201201250 (PMC3491838; doi:10.1002/emmm.201201250)

Supporting Information – Bajrami *et al*

Synthetic lethality of PARP and NAMPT inhibition in triple negative breast cancer cells

Table 1S of Supporting Information – page 2
Composition of siRNA library consisting of 44 PARPs and proteins involved in β-NAD^+ metabolism.

Table 2S of Supporting Information– page 3
Drug Effect Z scores. Gene symbols for each siGENOME SMARTPool are shown as are DE Z scores.

Table S3 of Supporting Information– page 4
PARP inhibitor sensitivity in a panel of triple negative cell lines.
Cells were plated in six-well plates and treated for 14 days, after which SFs were estimated.
Fold change survival under different conditions is shown in comparison to controls for each experiment.
p values shown were calculated using ANOVA.

Table S4 of Supporting Information– page 5
Inhibition of NAMPT using an inhibitor, FK866 sensitizes a panel of triple negative cell lines to olaparib.
Fold change has been calculated in comparison to olaparib treatment alone.
p values shown were calculated using ANOVA.

Table S5A of Supporting Information – page 6
In vivo efficacy of olaparib in combination with FK866 inhibitor in triple negative breast cancer xenografts. Repeated measures ANOVA *p* values of each comparison made in the *in vivo* study using a Newman-Keuls post test. Here, a repeated measures ANOVA analysis was used to compare tumour volumes in different cohorts across all time points.

Table S5B of Supporting Information – page 6
Mean tumour volumes and SEMs of tumour volumes for each cohort described in Figure 6.

Table S6 of Supporting Information – page 7
Student's t test analysis of Supporting Figure S2

Supporting Figure 1. – page 8
HTS analysis. Raw Cell Titre Glo luciferase readings from the HTS were processed as described in (Lord et al, 2008).
In brief, Cell Titre Go readings were first $\log_2$ transformed and then centered according to the plate $\log_2$ transformed median.
The effect on PARP inhibitor sensitivity caused by each siRNA was calculated according to the following equation:
Drug Effect (DE) of siRNA for gene *X* = (median centered data from olaparib-treated wells for gene *X*) - (median centered data from DMSO-treated wells for gene *X*). Calculation of the Median Absolute Deviation (MAD) was used to estimate the variance of the DE data. Standardised Z scores for DE were calculated using DE values, the median DE from the entire library and the MAD as variables. A similar analysis was conducted to estimate the effect of each siRNA upon cell viability in the absence of olaparib and siRNAs that caused significant cell inhibition alone (Z<-3) were excluded from the final analysis. In total we used data from three biological replicate screens in the final analysis. (A) Dynamic range of the screen/tranfection efficiency was assessed by comparing the median viability effects (and variance) of siRNAs targeting essential genes. Z prime (Boutros et al, 2006) plots generated by Cell HTS2 (Boutros et al, 2006) indicate a suitable dynamic range between lethal siRNAs targeting either PLK1 or UBB and control, non-targeting siRNAs and thus a high-efficiency transfection. (B) Schematic of high-throughput siRNA screen targeting 44 PARP and NAD metabolism proteins. The genes targeted in the siRNA library were curated from literature descriptions of proteins having well-established or casual links to NAD metabolism and these are listed in Supporting Table 1. CAL51 cells were reverse transfected in a 96 well-plate format with Dharmacon siGENOME SMARTPools siRNAs. Forty-eight hours after transfection, media either containing the clinical PARP1/2 inhibitor olaparib (1 μM final concentration) or the drug vehicle, DMSO, was added to replica plates. After five days continuous culture in the presence of olaparib, cell viability was estimated using Cell-Titre Glo (Promega). Screen data was processed as described in the Materials and Methods. (C) Performance of biological replicas analysed using Spearman's coefficient R= 0.45-0.97.

Supporting Figure 2. – page 9
Effect on cell viability of FK866 in (A) CAL51 (B) Hela (C) MDAMB468, (D) SUM149 (E) MDAMB231 (F) HS578T (G) BT20 and (H) DLD1 *BRCA2*$^{-/-}$ and *BRCA2* $^{+/+}$ cells. See also Supporting Table S6.

Supporting Figure 3. – page 10
(A) Effect of the FK866/olaparib combination on MCF10A cell viability. (B) Effect of FK866 on MCF10A cell viability. Cells were treated as in Figure 4. (C) Sensitising effect of NAMPT siRNA on olaparib sensitivity in *BRCA2*$^{-/-}$ or *BRCA2* $^{+/+}$ DLD1 cells. Viability was measured after six days of continuous exposure. Error bars represent the SEM from three independent experiments. **p*<0.001 (Student's t-test).

Supporting Figure 4. γH2AX focus formation – pages 11 - 15
(A) Confocal images of γH2AX foci (red), and nuclei (stained with DAPI – blue) are shown. The scale bar represents 10 μm. (B) Effect of FK866 on caspase 3/7 activity as measured by the ApoToxGlo Triplex kit (Promega). CAL51 cells were exposed to FK866 for 48 hours and assessed as in the materials and methods.

Supporting Table 1

Symbol	Gene ID	Accession
**PARPs**		
PARP1	142	NM_001618
PARP2	10038	NM_001042618
PARP3	10039	NM_001003931
PARP4	143	NM_006437
TNKS	8658	NM_003747
TNKS2	80351	NM_025235
PARP6	56965	NM_020214
TIPARP	25976	NM_015508
PARP8	79668	NM_024615
PARP9	83666	NM_031458
PARP10	84875	NM_032789
PARP11	57097	NM_020367
PARP12	64761	NM_022750
PARP13	56829	NM_020119
PARP14	54625	NM_017554
PARP15	165631	NM_152615
PARP16	54956	NM_017851
**PARG**		
PARG	8505	NM_003631
**SIRTs**		
SIRT1	23411	NM_012238
SIRT2	22933	NM_030593
SIRT3	23410	NM_012239
SIRT4	23409	NM_012240
SIRT5	23408	NM_031244
SIRT6	51548	NM_016539
SIRT7	51547	NM_016538
**ADP-ribose synthases**		
CD38	952	NM_001775
BST1	683	NM_004334
**ADP-ribose transferases**		
ART1	417	NM_004314
ART3	419	NM_001179
ART4	420	NM_021071
ART5	116969	NM_053017
**ADP-Ribosylhydrolases**		
ADPRHL1	113662	NM_138430
ADPRHL2	54936	NM_017825
ADPRH	141	NM_001125
NUDT5	11164	NM_014142
NUDT9	53343	NM_024047
NUDT14	256281	NM_177533
NUDT12	83594	NM_031438
**Nicotinamide nucleotide adenyl transferases**		
NMNAT1	64802	NM_022787
NMNAT2	23057	NM_015039
NMNAT3	349565	NM_178177
**Nicotinamide phophoribosyltransferase**		
NAMPT	10135	NM_005746
**NAD synthetase**		
NADSYN1	55191	NM_018161
**Nicotinic acid phophoribosyltransferase**		
NAPRT	93100	NM_145201

Supporting
Table 2

siRNA SMARTpool	Median PARPi Z-score Sensitivity	Median Growth % siCONTROL
NAMPT	-9.87	98
PARP10	-1.55	105
BST1	-1.09	102
NMNAT3	-0.88	101
PARP3	-0.79	100
PARP11	-0.72	100
PARP4	-0.66	99
PARP6	-0.65	101
NUDT9	-0.59	105
TNKS2	-0.51	100
PARP15	-0.46	103
NAPRT	-0.44	98
PARP2	-0.41	99
ART5	-0.40	103
ADPRH	-0.40	105
PARP12	-0.36	103
TIPARP	-0.29	100
SIRT1	-0.26	102
NADSYN1	-0.23	101
ART3	-0.22	102
ART1	-0.22	99
TNKS	-0.19	100
PARP14	-0.15	104
CD38	-0.15	101
NUDT14	-0.13	103
SIRT7	-0.02	100
PARP13	-0.01	100
SIRT5	0.02	99
PARP9	0.11	96
NUDT5	0.16	102
ADPRHL1	0.20	103
PARP16	0.21	103
ADPRHL2	0.22	104
NMNAT1	0.26	104
NUDT12	0.29	103
PARP1	0.31	98
PARP8	0.32	98
SIRT6	0.33	100
ART4	0.34	102
SIRT3	0.34	97
SIRT4	0.35	99
SIRT2	0.42	98
PARG	0.51	99
NMNAT2	0.72	101

Supporting Table 3

A

Cell line	SF_{50} [Olaparib] µM
**HS578T**	6.3418
**MDA-MB-231**	26.7178
**SUM149**	0.0133
**MDA-MB-468**	1.2515
**MDA-MB-436**	0.0002
**CAL51**	1.0140
**BT20**	>100

B

CAL51

siRNA	SF_{50} nM	Fold increase *vs.* siCON1 transfected cells	ANOVA p value *vs.* siCON1
**NAMPT siRNA 1**	96	8	< 0.0001
**NAMPT siRNA 2**	26	29	< 0.0001
**NAMPT siRNA 3**	84	9	< 0.0001
**NAMPT siRNA SmartPool**	75	10	< 0.0001
**siCON 1**	758	1	ns
**BRCA2 siRNA**	1	661	< 0.0001

C

HeLa

siRNA	SF_{50} nM	Fold increase *vs.* siCON1 transfected cells	ANOVA p value *vs.* siCON 1
**NAMPT siRNA 1**	468	8	< 0.0001
**NAMPT siRNA 2**	1041	3	0.0001
**NAMPT siRNA 3**	256	14	< 0.0001
**NAMPT siRNA SmartPool**	128	28	< 0.0001
**siCON 1**	3631	1	ns
**BRCA2 siRNA**	6	634	< 0.0001

D

CAL51

Treatment	SF_{50} nM	Fold increase *vs.* olaparib alone	ANOVA p value *vs.* olaparib alone
**Olaparib alone**	2377	1	ns
**FK866i 10^{-13}+ Olaparib**	2047	1	ns
**FK866i 10^{-12} + Olaparib**	1667	1	ns
**FK866i 10^{-11} +Olaparib**	1895	1	ns
**FK866i 10^{-10} +Olaparib**	860	3	0.0006
**FK866i 10^{-9} + Olaparib**	713	3	< 0.0001
**FK866i 10^{-8} + Olaparib**	442	5	< 0.0001
**FK866i 10^{-7} + Olaparib**	67	36	< 0.0001

E

HeLa

Treatment	SF_{50} nM	Fold increase *vs.* olaparib alone	ANOVA p value *vs.* olaparib alone
**Olaparib alone**	7792	1	ns
**FK866i 10^{-13}+ Olaparib**	2156	4	< 0.0001
**FK866i 10^{-12} + Olaparib**	4241	2	< 0.0001
**FK866i 10^{-11} +Olaparib**	3774	2	< 0.0001
**FK866i 10^{-10} +Olaparib**	4100	2	0.0001
**FK866i 10^{-9} + Olaparib**	4916	2	0.0051
**FK866i 10^{-8} + Olaparib**	2913	3	< 0.0001
**FK866i 10^{-7} + Olaparib**	675	12	< 0.0001

Supporting Table 4

A

MDAMB468

Treatment	SF_{50} nM	Fold increase *vs.* olaparib alone	ANOVA p value *vs.* olaparib alone
**Olaparib alone**	982	1	ns
**FK866i 10^{-11} +Olaparib**	269	4	< 0.0001
**FK866i 10^{-10} +Olaparib**	164	6	0.0001
**FK866i 10^{-9} + Olaparib**	10	95	0.0051

B

SUM149

Treatment	SF_{50} nM	Fold increase *vs.* olaparib alone	ANOVA p value *vs.* olaparib alone
**Olaparib alone**	141	1	ns
**FK866i 10^{-11} +Olaparib**	16	9	ns
**FK866i 10^{-10} +Olaparib**	4	39	0.0127
**FK866i 10^{-9} + Olaparib**	1	97	0.0012

C

MDAMB231

Treatment	SF_{50} nM	Fold increase *vs.* olaparib alone	ANOVA p value *vs.* olaparib alone
**Olaparib alone**	11385	1	ns
**FK866i 10^{-11} +Olaparib**	6228	2	0.053
**FK866i 10^{-10} +Olaparib**	4901	2	0.0002
**FK866i 10^{-9} + Olaparib**	5281	2	0.0001

D

HS578T

Treatment	SF_{50} nM	Fold increase *vs.* olaparib alone	ANOVA p value *vs.* olaparib alone
**Olaparib alone**	5525	1	ns
**FK866i 10^{-11} +Olaparib**	1055	5	0.012
**FK866i 10^{-10} +Olaparib**	956	6	0.0002
**FK866i 10^{-9} + Olaparib**	1343	4	0.0077

E

BT20

Treatment	SF_{50} nM	Fold increase *vs.* olaparib alone	ANOVA p value *vs.* olaparib alone
**Olaparib alone**	11371	1	ns
**FK866i 10^{-11} +Olaparib**	2773	4	ns
**FK866i 10^{-10} +Olaparib**	1571	7	0.0068
**FK866i 10^{-9} + Olaparib**	720	16	0.0002

F

				**DLD1 BRCA2 $^{+/+}$**		**DLD1 BRCA2 $^{-/-}$**	
**Cell Line**	**Treatment**	**SF_{50} nM**	**Fold increase *vs.* olaparib alone**	**ANOVA p value *vs.* olaparib alone**	**ANOVA p value *vs.* FK866i 10^{-7} + Olaparib**	**ANOVA p value *vs.* olaparib alone**	**ANOVA p value *vs.* FK866i 10^{-7} + Olaparib**
**DLD1 BRCA2 $^{+/+}$**	**Olaparib alone**	1192.4	1	ns	ns	0.0003	< 0.0001
	**FK866i 10^{-7} + Olaparib**	869.4	1	ns	ns	< 0.0001	< 0.0001
**DLD1 BRCA2 $^{-/-}$**	**Olaparib alone**	1.7	1	0.0003	< 0.0001	ns	0.0007
	**FK866i 10^{-7} + Olaparib**	0.4	4	< 0.0001	< 0.0001	0.0007	ns

Supporting
Table 5

A

	Significant?	Summary
Olaparib/FK866 combination vs Vehicle	p<0.001	***
Olaparib/FK866 combination vs FK866	p<0.05	*
Olaparib/FK866 combination vs Olaparib	p<0.05	*
Olaparib vs Vehicle	p>0.05	ns
Olaparib vs FK866	p>0.05	ns
FK866 vs Vehicle	p>0.05	ns

B

	**Vehicle**		**15 mg/kg Olaparib**		**6 mg/kg FK866**		**Olaparib/FK866 combination**	
Days	Mean	SEM	Mean	SEM	Mean	SEM	Mean	SEM
0	1.00	0.00	1.00	0.00	1.00	0.00	1.00	0.00
3	1.33	0.14	1.67	0.19	1.72	0.30	1.42	0.23
6	1.73	0.16	2.02	0.21	2.56	0.47	2.68	0.39
9	2.37	0.35	2.50	0.36	2.64	0.38	2.15	0.32
12	2.97	0.33	4.07	0.68	3.50	0.54	3.00	0.39
15	3.22	0.41	3.11	0.47	3.43	0.70	3.88	0.65
18	5.13	0.67	5.95	1.19	6.02	0.91	4.85	0.76
21	6.86	0.69	6.84	1.58	6.55	1.06	5.50	1.14
24	9.14	1.46	9.45	2.16	8.25	1.71	6.92	1.71
27	12.83	2.53	11.49	2.25	11.55	2.29	7.75	2.22
30	13.48	3.73	8.57	1.55	10.03	2.03	7.92	2.54
33	14.32	3.05	9.99	1.57	12.92	3.35	7.64	2.70
36	21.05	6.01	11.71	2.01	13.26	3.24	9.13	3.41
39	22.73	6.68	19.05	3.08	17.93	5.43	10.94	4.11

Supporting
Table 6

Cell line	FK866 concentration (M)	Student's t test p value vs. vehicle
CAL51	1 x 10E-13	>0.05
CAL51	1 x 10E-12	>0.05
CAL51	1 x 10E-11	>0.05
CAL51	1 x 10E-10	>0.05
CAL51	1 x 10E-9	>0.05
CAL51	1 x 10E-8	>0.05
CAL51	1 x 10E-7	<0.05
HeLa	1 x 10E-13	>0.05
HeLa	1 x 10E-12	>0.05
HeLa	1 x 10E-11	>0.05
HeLa	1 x 10E-10	>0.05
HeLa	1 x 10E-9	>0.05
HeLa	1 x 10E-8	>0.05
HeLa	1 x 10E-7	>0.05
MDAMB468	1 x 10E-11	>0.05
MDAMB468	1 x 10E-10	<0.05
MDAMB468	1 x 10E-9	<0.05
SUM149	1 x 10E-11	>0.05
SUM149	1 x 10E-10	>0.05
SUM149	1 x 10E-9	>0.05
MDAMB231	1 x 10E-11	>0.05
MDAMB231	1 x 10E-10	>0.05
MDAMB231	1 x 10E-9	<0.05
HS578T	1 x 10E-11	>0.05
HS578T	1 x 10E-10	>0.05
HS578T	1 x 10E-9	>0.05
BT20	1 x 10E-11	>0.05
BT20	1 x 10E-10	>0.05
BT20	1 x 10E-9	<0.05
DLD1 BRCA2+/+	1 x 10E-7	>0.05
DLD1 BRCA2-/-	1 x 10E-7	>0.05

Supporting Figure 1

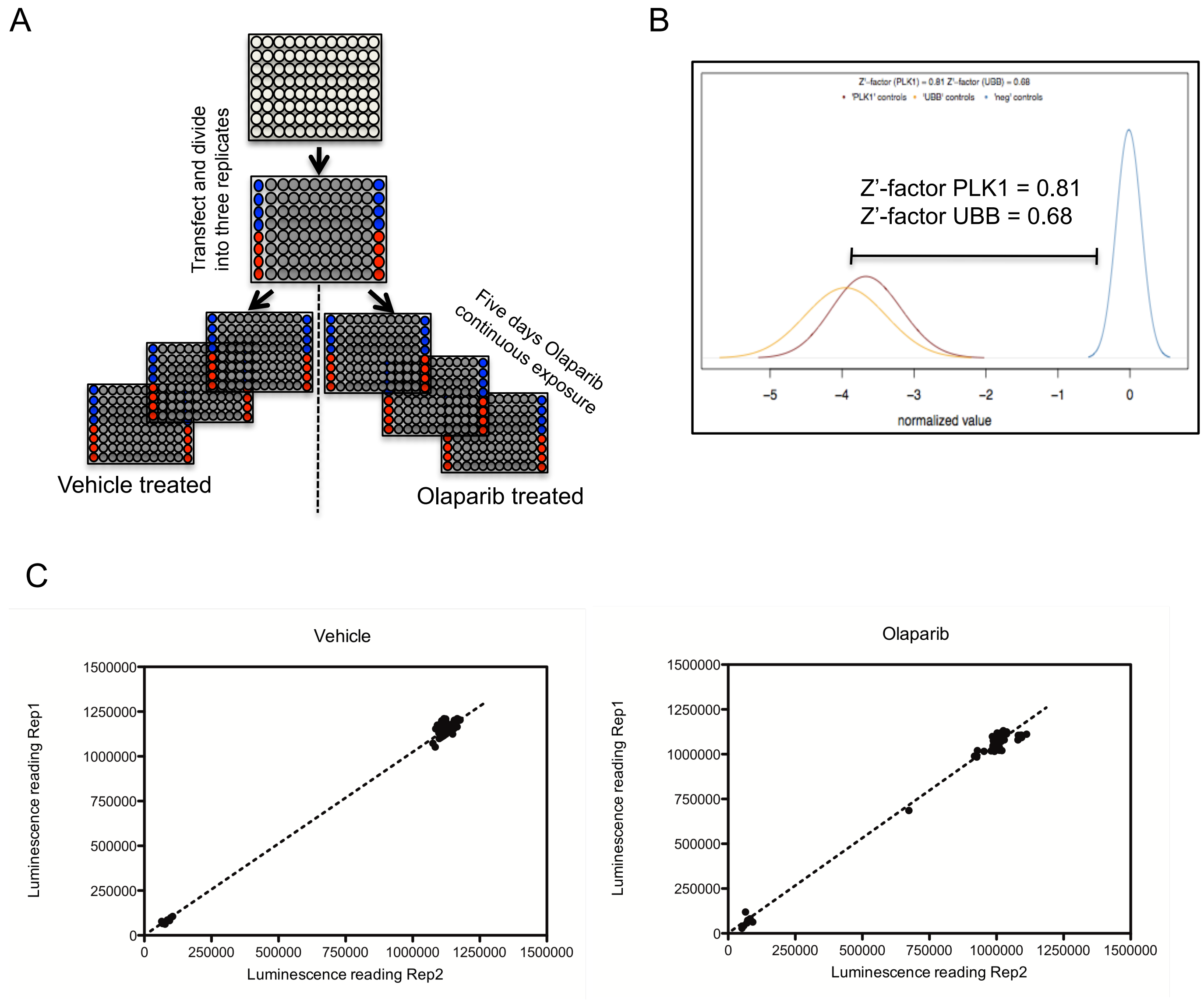

Vehicle	rep1 vs rep2	rep1 vs rep3	rep2 vs rep 3
Spearman R	0.6401	0.9674	0.4507

Olaparib	rep1 vs rep2	rep1 vs rep3	rep2 vs rep 3
Spearman R	0.7338	0.9963	0.5897

Supporting
Figure 2

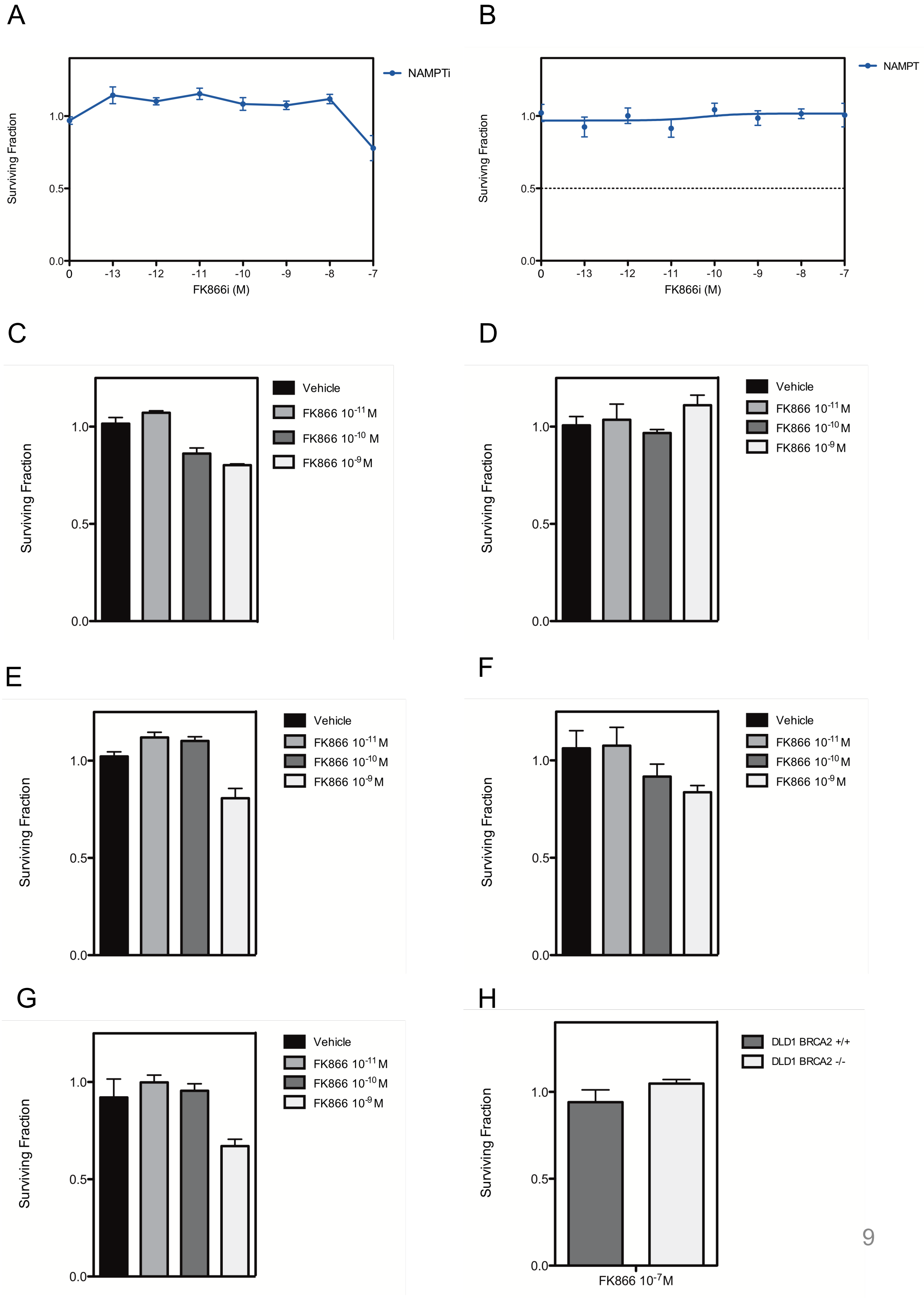

Supporting
Figure 3

A

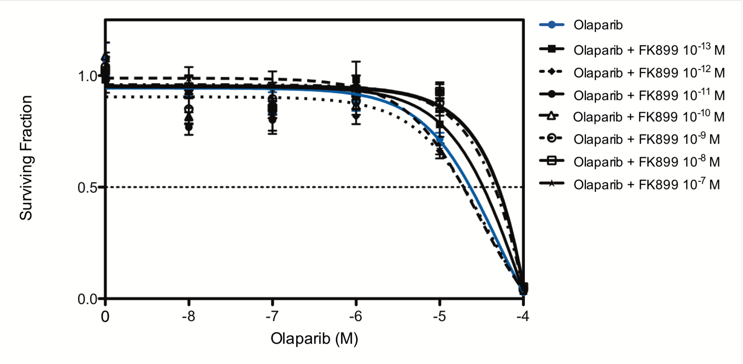

B

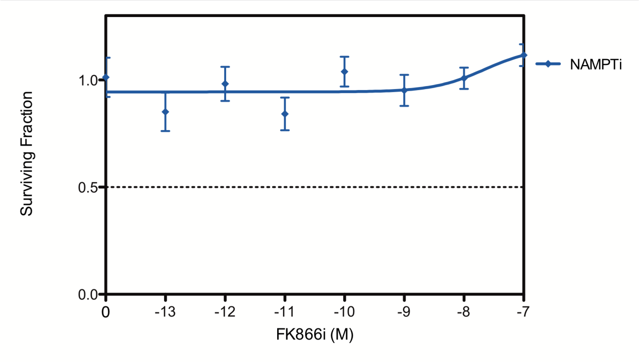

C

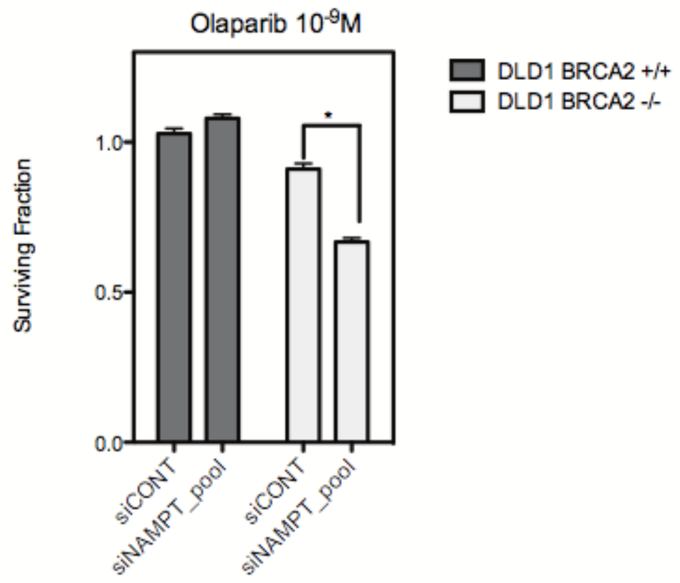

Supporting
Figure 4

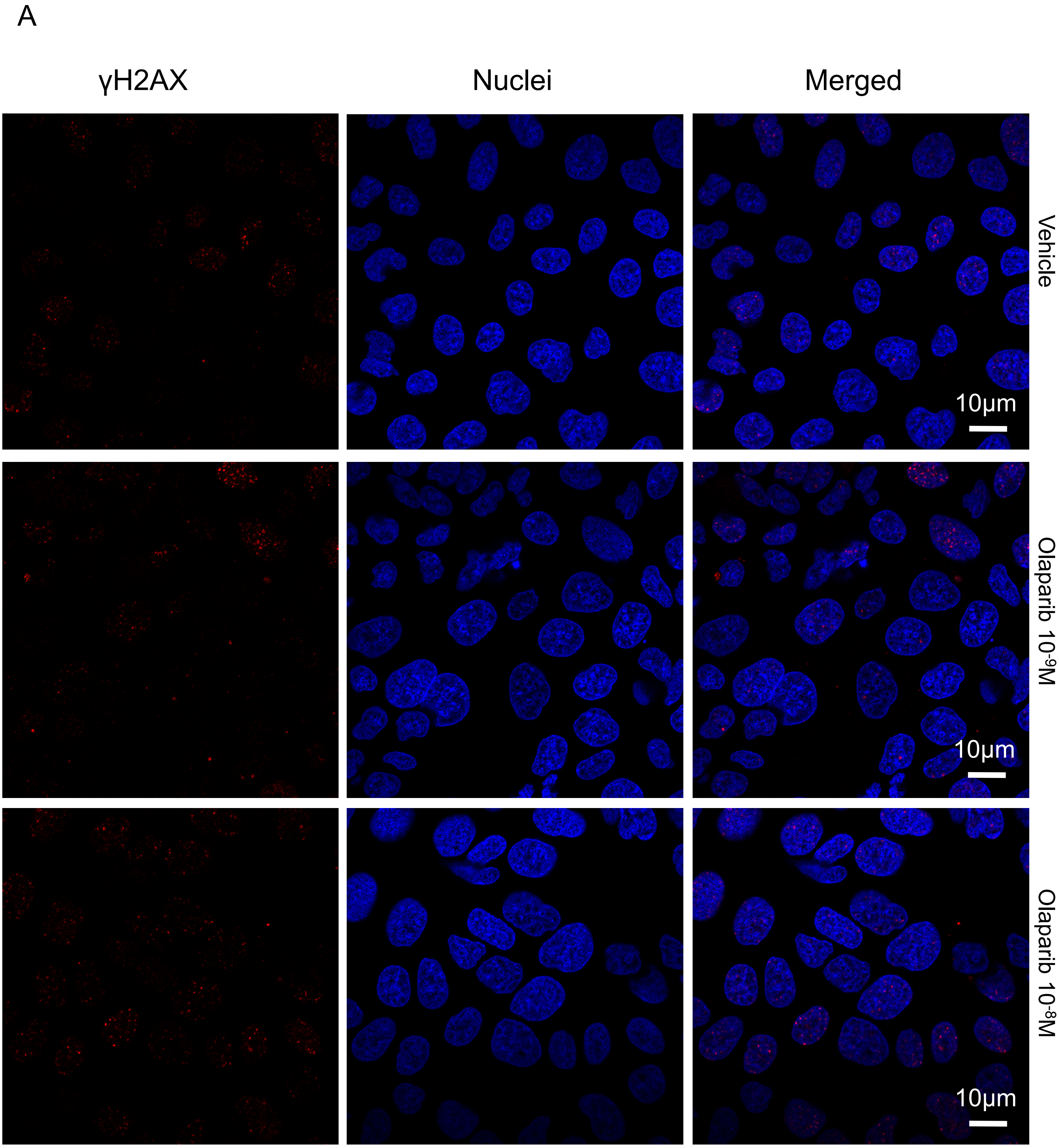

Supporting Figure 4

A

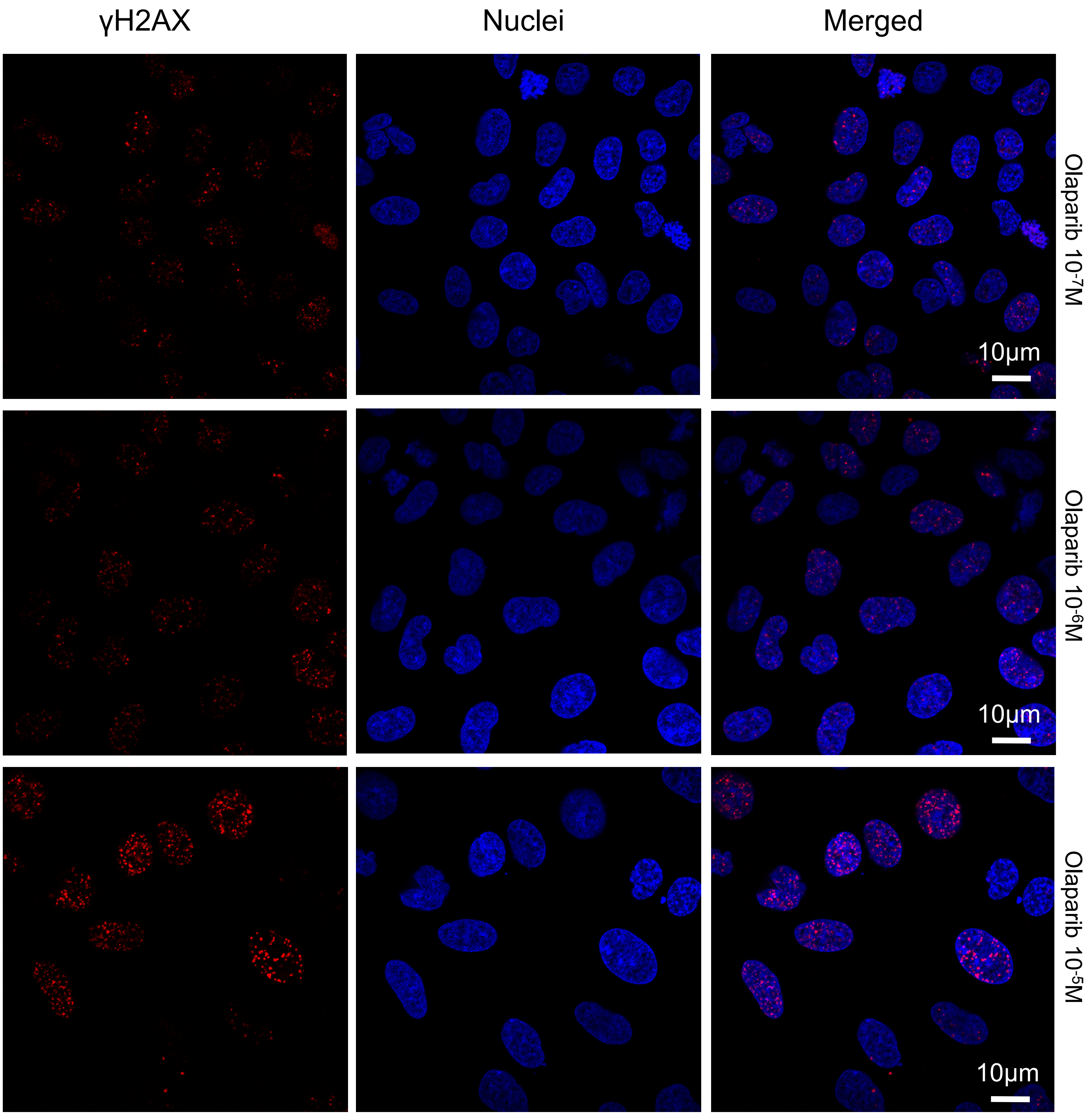

Supporting
Figure 4
A

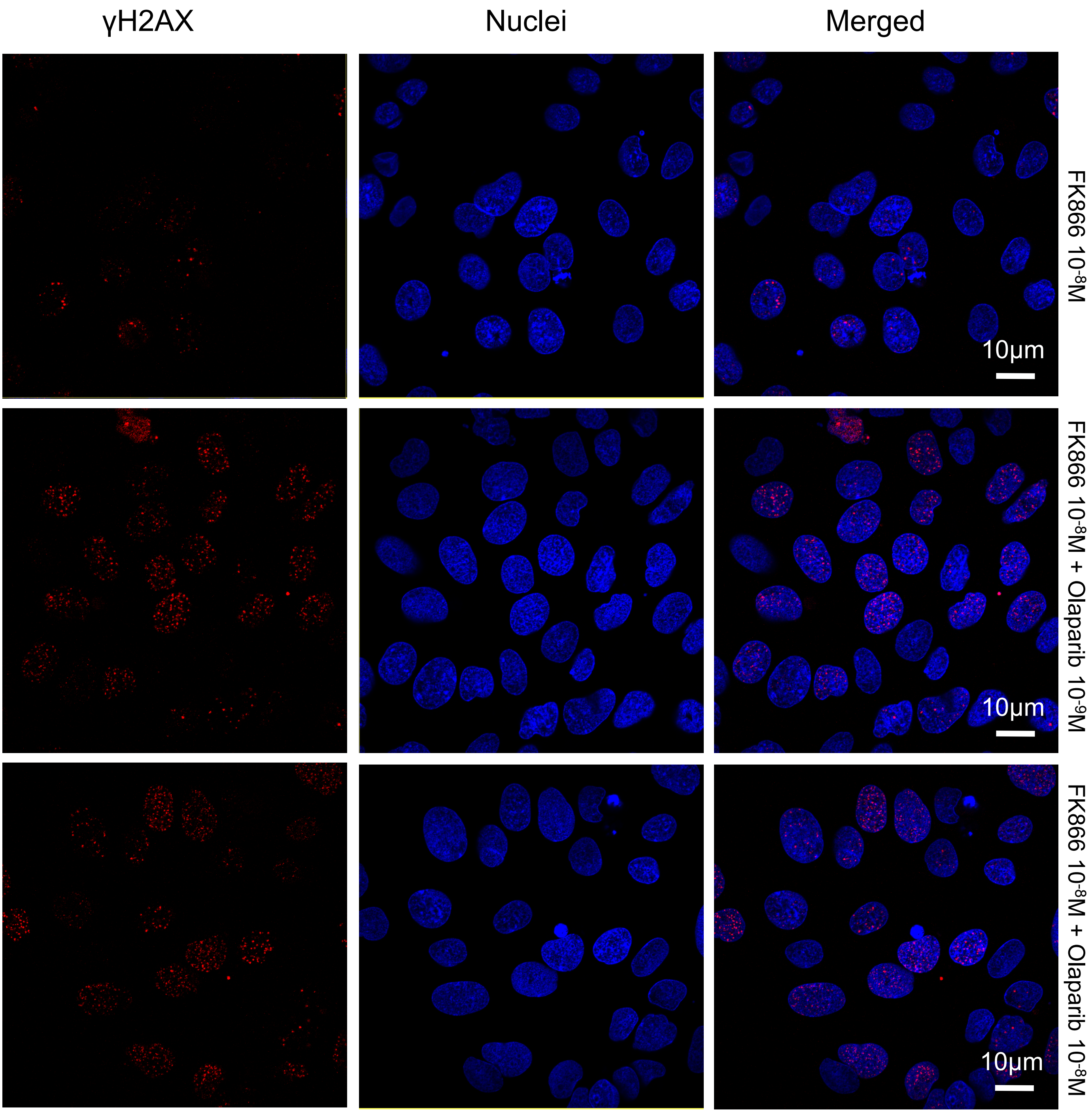

Supporting
Figure 4
A

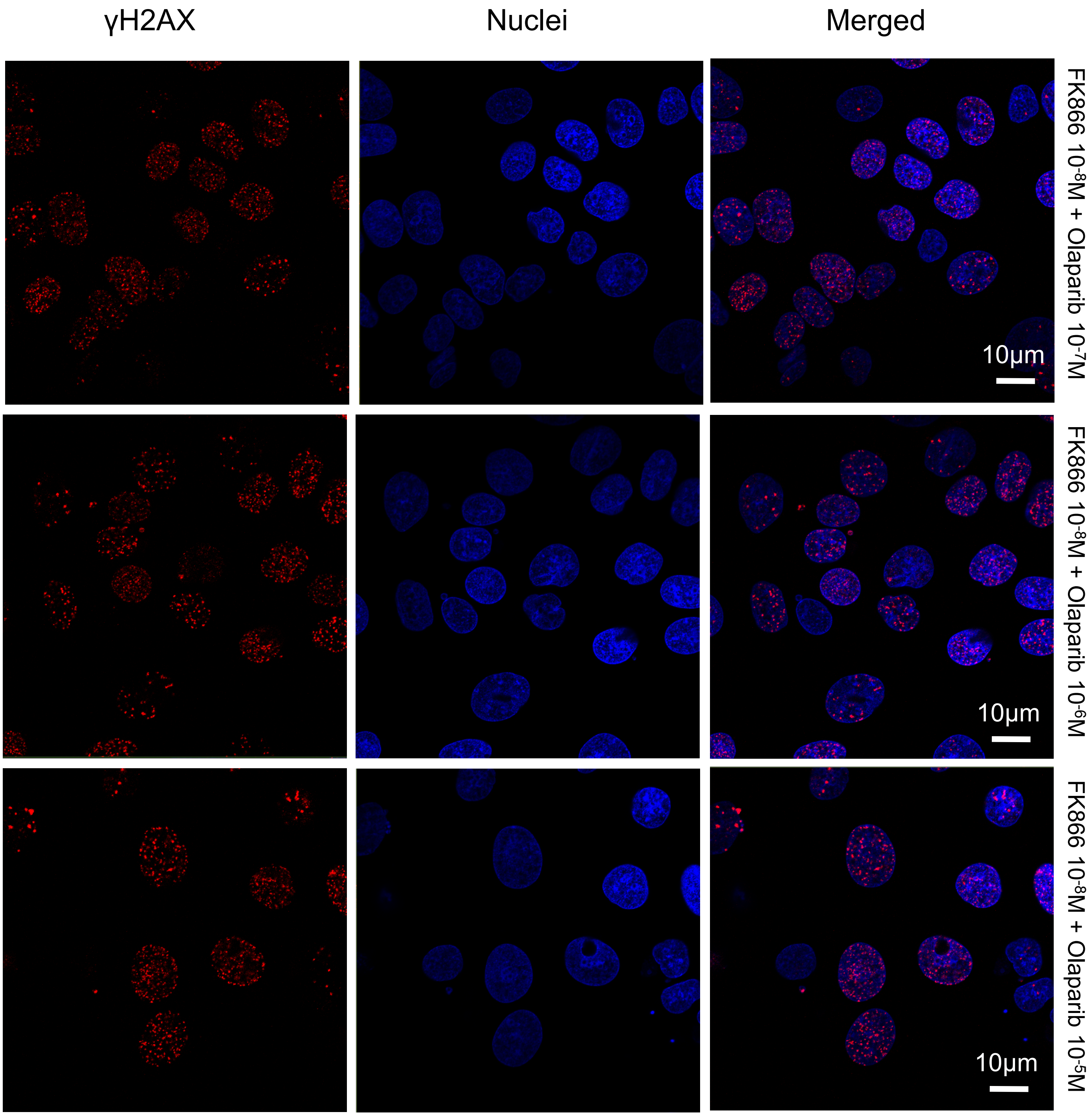

Supporting
Figure 4

B

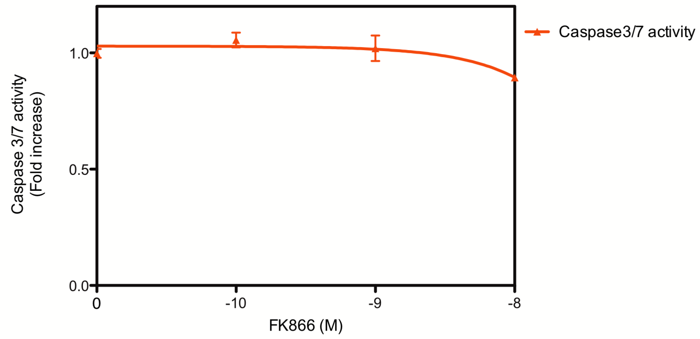

Supplement: Supplementary file 2 [file emmm0004-1087-SD2.pdf]
